# Combined Point Mutation in a Single Allele of the TP53 Gene Potentiates the Oncogenic and Transcriptional Properties of TP53 in Lung Cancer

**DOI:** 10.7150/ijbs.131687

**Published:** 2026-09-10

**Authors:** Sicheng Bian, Huiqin Bian, Hiroaki Koyama, Dmytro Kompaniiets, William Tse, Mithun V Shah, Aref Al-Kali, Alexander Miron, Bin Liu, Shujun Liu

**Affiliations:** 1 Department of Medicine, The MetroHealth System, Case Western Reserve University, 2500 Metro Health Drive, Cleveland, OH 44109, USA.; 2 Hormel Institute, University of Minnesota 801 16th Ave NE, Austin, MN 55912, USA.; 3 Gene and Cell Therapy Institute, The MetroHealth System, Case Western Reserve University, 2500 MetroHealth Drive, Cleveland, OH 44109, USA.; 4 Division of Hematology, Mayo Clinic, 200 1st St SW, Rochester, MN 55901, USA.; 5 Department of Genetics and Genome Sciences, Case Western Reserve University Medical School, 2109 Adelbert Road, Cleveland, OH 44106, USA.

**Keywords:** DNA-binding affinity, Lung cancer metastasis, Tumor microenvironment, Cytokine regulation, Chemoresistance

## Abstract

Although multi-hit TP53 mutations correlate with rapid disease progression, treatment resistance, recurrence, and poor survival, their occurrence within an individual TP53 gene molecule (cbTP53) remains unexplored. Consequently, the impact of cbTP53 on protein conformation, transcriptional architecture, and oncogenicity is unknown. We show that cbTP53 with combined mutations (Y220C+R248Q+R282Q) does not drastically alter local stability or structural conformation, but significantly affects TP53 binding to DNA promoters comparing it to a single Y220C mutation. Functionally, cbTP53 promotes divergent cellular phenotypes, characterized by enhanced cell proliferation, increased formation of multicellular tumor spheroids (MCTS) and spheroids, altered responses to chemotherapeutic drugs (e.g., cisplatin, carboplatin) and p53 inhibitors (e.g., MB710, PK11007), and increased resistance to carboplatin-induced DNA damage. Furthermore, cbTP53 is linked to distinct immune cell profiles (e.g., CD4+ and CD8+ T cells, nature killer cells, dendritic cells), more circulating tumor cells, a higher rate of tumor engraftment, and an increased number of lung tumor nodules. Mechanistically, introduction of R248Q/R282Q mutations changes 42.7% of Y220C-regulated gene expression, and cbTP53 (vs Y220C) owns a distinctive transcriptomic signature with 1,004 up- and 1,747 down-regulated genes. cbTP53 shows the enhanced binding affinity in the promoters of inflammatory cytokines, leading to their increased production. This study establishes causative and mechanistic links of cbTP53 to lung aggressive cancer metastasis and disease progression, providing a novel perspective on the genetics of TP53 mutations in cancer.

## Introduction

Mutations in *TP53* tumor suppressor gene are found in over 50% of all human cancers. TP53 mutations mainly include missense, truncating, inframe, and splice mutations resulting in the loss of tumor suppressor activities with gain-of-function properties and promotion of tumorigenesis 1. But missense mutations are predominant (~80%), which occur within the central sequence-specific DNA binding domain (DBD) with single amino acid substitutions, including six “hotspot” codons (R175, R213, G245, R248, R273, R282). This accounts for ~25% of all *TP53* mutations 2. Outside the DNA-binding surface of TP53, Y220C is the most common cancerous mutation 3. This mutation reduces protein thermostability leading to protein aggregation at physiological temperatures. The “hotspot” mutants, including the Y220C mutant, comprise up to 25% of all p53 mutations in total 4. As a single mutational event, the impacts of the above TP53 mutations on p53 DBD structure, stability and dynamics have been well-investigated, but a comprehensive understanding of the impacts of multiple TP53 mutations ("multi-hits") on driving cancer pathogenesis and drug resistance remains less understood.

Non-small cell lung cancer (NSCLC), comprising 85% of lung cancers, is a leading cause of cancer-related death globally. Despite advances in treatment modalities like surgery, chemotherapy, radiation, and targeted therapies, poor prognosis and disease recurrence persist due to drug resistance and complex genetic alterations. Among these genetic aberrations, *TP53* is one of the most mutated genes 5, 6 and the most extensively investigated prognostic marker in NSCLC. *TP53* mutations appear in over 50% of patients with NSCLC 7-9, who have poorer clinical outcomes and shorter survival time than those without mutations 10-12. Importantly, multi-hit *TP53* mutations are associated with more aggressive disease and worse prognosis compared to single-hit mutations (monomutations) 13, 14. However, it remains a mystery whether such multiple mutations occur within an individual *TP53* gene molecule (cbTP53), and if so, whether and how cbTP53 affects, in a different way, the TP53 protein structure, DNA binding affinity, transcriptional gene regulation and tumor cell behaviors. To address these questions, we utilized multiple approaches encompassing structural biology techniques and experimental validation both *in vitro* and *in vivo*. Our investigation demonstrated that, in contrast to monomutations, cbTP53 profoundly modifies TP53 functions, influencing lung cancer cellular/molecular plasticity and the *in vivo* tumor microenvironment.

## Materials and Methods

### Structural analyses and comparisons

Structural analyses and comparisons were performed using ChimeraX software 15 alongside an extensive literature review. The structures were superimposed onto a single TP53 subunit, and RMSD values were calculated for comparison. Mutation sites and their surrounding regions were assessed and compared, and the interactions of the mutated residues with neighboring residues were mapped and analyzed. Mutagenesis modeling was conducted for the unavailable R248Q mutant structure. The mutated residue and the positions of its side chain were compared with those of the wild-type R248 side chain and its interactions.

### Reagents

The drugs and chemicals include cisplatin (Sigma, #1134357), and caborplatin (Sigma, #1096407), etoposide (Research Products International Corp, #E55500-0.1). For *in vitro* treatment, cisplatin, etoposide and carboplatin were dissolved in dimethyl sulfoxide (DMSO) and stored at -80 °C.

### Cell lines and cell culture

LLC/2 cells were newly purchased from American Type Culture Collection with no further authentication or testing for mycoplasma. Cell lines were grown in DMEM (GE Healthcare, #SH30027.01) supplemented with 10% fetal bovine serum (FBS, Gibco by Life Technologies^TM^, #16140-071) and Antibiotic-Antimycotic (Gibco by Life Technologies^TM^, #15240062) at 37 °C under 5% CO_2_. No cell line used in this paper is listed in the database of commonly misidentified cell lines maintained by ICLAC (International Cell Line Authentication Committee).

### *TP53* mutation constructs

Mutations (Y220C, R248Q, R282Q) on TP53 gene (MigR1-TP53) were created using the QuikChange Site-Directed Mutagenesis Kits (Agilent, #200519) following manufacturer's instructions. All mutations were verified by DNA sequencing in GENEWIZ.

### Retrovirus vector, virus production, virus infection and GFP sorting

For virus production, HEK-293 (3.8 × 10^6^) cells were planted in a 10 cm cell culture dish for 24 hours, and transfected with 6 µg of targeted or scrambled plasmids (with GFP) using calcium phosphate transfection reagent (CalPhos™ Mammalian Transfection Kit, Sigma, #233-140-8), following the manufacture's instruction. The retroviruses were harvested at 48 and 72 hours after transfection and concentrated using the protocol of the Lenti-X™ Concentrator (Clotech, #631232). For virus infection, LLC/2 cells (1 × 10^6^) were infected by the retroviruses using Polybrene (final concentrate 4 µg/ml) in 1 ml medium. The GFP positive cells were sorted at 72 hours post-infection, expanded and sorted again to be over 95% purity for further investigations. Fluorescence images were captured on a Nikon Eclipse Ts2R microscope using an excitation wavelength of 475 nm.

### IncuCyte S3 live-cell analysis

LLC/2 cells with various TP53 mutations were seeded into a 12-well culture plates for 24 hours. The plates were incubated in the IncuCyte S3 Live-Cell Analysis System (Leica Microsystems) for real-time analysis. Data was analyzed by the software provided. The total area of phase for each picture was calculated, which represents the confluence of LLC/2 cells.

### CCK-8 assays

CCK-8 assays were performed using Cell Counting Kit-8 (CCK-8, Dojindo Molecular Technologies, #CK04-11). Briefly, LLC/2 cells (1.5 × 10^4^) in DMEM medium (100 µl) were dispensed into 96-well flat-bottomed microplates and drugs (if necessary) were added after 24 hours of incubation. The cells were cultured for an additional 24 or 48 hours, and CCK-8 reagent (10 µl) was added to each well. Then the microplates were incubated at 37 °C for another 1~2 hours. Absorbance was measured at 450 nm using a microplate reader and the results were expressed as a ratio of three TP53 to one or no mutations with or without chemodrug treatment (as 100%). Four wells were sampled per experimental group in each experiment.

### Multicellular tumor spheroid (MCTS)-forming assays

About 200 LLC/2 cells were seeded in appropriate medium in each well of a Corning® Costar® Ultra-Low attachment plate (Corning, #3473), and incubated up to 10 days. During the incubation, the cells were pipetted several times to prevent cell aggregates. Then the number of MCTSs was counted and images were taken.

### Clonal matrix-embedded sphere-forming assays

About 200 cells were mixed with 50 µl of Matrigel and grew in 24-well plates containing 1.0 ml 5% FBS medium. Seven days after seeding, spheroid digital images were captured in 10× magnification at a resolution of 5184 × 2912 pixels. The captured images were imported into Fiji (Image J) and analyzed for size on days 5 and 7. A freehand selection tool was used to trace the outer periphery of each spheroid, and the area enclosed by the traced control was calculated using Fiji's integrated measurement tools. The area was quantified in square pixels (pixels^2). Results are presented as a mean and standard deviation of a single well, with each well containing 3 structures for diameter measurement.

### Alkaline comet assay (single-cell gel electrophoresis)

LLC/2 cells with Y220C or cbTP53 mutations were treated with IC_50_ values of chemodrugs for 24 and 48 hours, and subjected to Alkaline comet assays (Cell Biolabs Inc; OxiSelect™ Comet Assay Kit, #STA-351) following manufacturer's instructions (http://casplab.com/). The images were quantified and calculated using software CASPlab (CASP, Comet Assay Software Project). Olive Tail Moment = Tail DNA% × Tail Moment Length; Tail Moment Length is measured from the center of the head to the center of the tail. The Tail Moment has been suggested to be an appropriate index of induced DNA damage in considering both the migration of the genetic material as well as the relative amount of DNA in the tail.

### Cytokine arrays

The LLC/2 cells carrying scramble, Y220C or cbTP53 were growing 72 hours in medium supplemented with exosome-depleted FBS. Then the production of cytokines in culture medium was determined using Proteome ProfilerTM Array (R&D System, Mouse Cytokine Array Panel A, #ARY006) following the manufacturer's instruction. Integrated density values were measured for each spot and normalized to positive controls on each membrane using Image J.

### RNA isolation, cDNA preparation and quantitative PCR (qPCR)

According to the Kit instructions, the total RNA was isolated using RNeasy Kit (QiaGen, #217004), and complementary DNA (cDNA) synthesis was performed using SuperScript® III First-Strand Synthesis System (Invitrogen, #18080-051). The expression of target genes was assessed by SYB Green qPCR (Applied Biosystems, #4309155). The levels of GAPDH were used as normalization and the expression of the targets was analyzed using the ΔCT approach. The primers used are listed in Supplementary Table 9.

### Western blotting

The whole cellular lysates were prepared by harvesting the cells in 1× cell lysis buffer (20 mM HEPES (pH 7.0), 150 mM NaCl and 0.1% NP40) supplemented with 1 mM phenylmethane sulfonyl fluoride (PMSF; Sigma #10837091001), 1× Phosphatase Inhibitor Cocktail 2 and 3 (Sigma #P5726, #P0044), and 1× protease inhibitors (protease inhibitor cocktail set III; Calbiochem-Novabiochem #539134). The proteins were resolved by sodium dodecyl sulfate (SDS)-polyacrylamide gel electrophoresis, transferred onto PVDF membranes (GE Healthcare #10600023), blocked by 5% non-fat milk followed by probing with primary anti-GFP (Novus, # NB600-308), human anti-P53 (Cell Signaling Technology, #2527S) or anti-β-Actin or the secondary antibody (goat anti-rabbit IgG, Cell Signaling Technology, 7076S; Anti-mouse IgG (H+L) (DyLight 680 Conjugate), Cell Signaling Technology, #5470S; Anti-rabbit IgG (H+L) (DyLight 800 4X PEG Conjugate, Cell Signaling Technology, # 5151S).

### Chromatin immunoprecipitation (ChIP)

ChIP assays were performed in LLC/2 cells with TP53 mutations using the EZ ChIP Assay Kit (EMD Millipore, #17-371RF) according to the manufacturer's standard protocol and P53 antibody against human TP53 (Cell Signaling Technology, #2527S). To analyze ChIP-qPCR product, we used “percent input” methods: %Input = 2^(Ct(Input)-Ct(ChIP))^ × dilution ration/100. The primers specifically for target gene promoters are listed in Supplementary Table 10.

### Establishment of metastatic lung cancer mouse model

C57BL/6 mice (6-8 weeks old, female) were purchased from the Jackson Laboratory. All animal procedures were performed according to NIH guidelines and approved by the Committee on Animal Care at the Case Western Reserve University. To establish metastatic lung cancer mouse model, 2 × 10^4^ LLC/2 cells expressing Y220C or cbTP53 were injected intravenously via the tail vein into syngeneic C57BL/6 mice (male, 4-6 weeks), mimicking the natural process of tumor cells entering the bloodstream and circulating to the lungs. Mice were monitored daily for signs of deteriorating health as indicated by weight loss, slow movement, or hunched posture. All mice had free access to food and water throughout the study. The mice were sacrificed 4 weeks after initial cell injection for further investigation.

### RNA sequencing and data processing

Total RNA was extracted using a RNeasy Mini Kit (Qiagen; Cat# 74104) and sequenced (triplicate sample) at the Genomic Center Case Western Reserve University. In brief, RNA samples were quantified using a Qubit Flourometer (Invitrogen) and quality was determined using a Fragment Analyzer 5200 (Agilent). All samples used have a minimum RIN score of 7. rRNA was then depleted using NEBNext rRNA kit v2 (New England Biolabs). RNAseq Libraries were then prepared using the NEBNext Ultra II Directional RNA Library Prep kit (New England Biolabs). Final libraries were validated on the Fragment Analyzer, quantified via qPCR, and pooled at equimolar ratios. Libraries were then diluted, denatured and loaded onto the NovaSeq X-PLUS sequencing system (Illumina) for a single read 75 cycle run. BCL to FastQ and sample demultiplexing were performed on instruments using DRAGEN BCL Convert version 4.3.13. Data quality was also assessed using FastQC version 0.12.1 and Fastq Screen version 0.15.3. Data analysis was performed using CLC Genomics Workbench 25 (Qiagen) using first the RNA-seq Analysis workflow to map reads to the Mus musculus (GRCm39) genome and calculate TPM expression values. Triplicate data sets were compared using the Differential expression for RNA-seq workflow to generate significant differences in gene expression based on Bonferroni statistical analysis.

The six GO categories (biological processes, environmental information processing, cellular components, genetic information processing, human disease, metabolism, and organismal system) were analyzed using the R package clusterProfiler, with a Benjamini-Hochberg multiple testing adjustment and a false discovery rate cut-off of 0.05, using all expressed/detected genes as background control. Results are visualized as dotplots using clusterProfiler and ggplot2 R packages. Top GO down- or up-regulated categories were selected according to *P*-values (< 0.05) and illustrated as number of genes up- or down- regulated in the respective categories.

To identify and visualize gene groups associated with the TP53 mutation group, Venn diagrams were generated using the InterVenn tool (https://asntech.shinyapps.io/intervene/). For further pathway-level analysis, gene set enrichment analysis (GSEA) was performed using the Hallmark gene sets to identify significantly enriched gene sets in the TP53 mutation group.

### Blood collection, immune cell staining and flow cytometry

The whole blood was collected into EDTA anticoagulant tubes (treated with 10 µl, 0.5M EDTA, pH 8.0) through a syringe containing a small amount of 0.5 M EDTA. The cell non-specific antigens were blocked by adding 1 µl/million cells of BD Pharmingen™ Purified Rat Anti-Mouse CD16/CD32 (Mouse BD Fc Block™, BD Biosciences, #553142) and incubating 10 minutes at room temperature. Then the targeted antibodies were directly added to the blocking tubes (without removal of blocking antibodies) and incubated at 4 °C in the dark. To lyse the red blood cells, 900 µl 1 × RBC lysis buffer (FACS Lysing Solution 10 ×, BD Biosciences, #349202) was added into each sample tube, mixed by vortex and incubated at room temperature in the dark for 15 minutes. The supernatant of hemolysis was discarded by centrifuge at 300 g, 4 °C for 10 minutes. The dead cells were stained by adding 100 µl of 1 × SYTOX BLUE [1 µl SYTOX BLUE (SYTOX BLUE DEAD CELL STAIN FOR, BD Biosciences, #501137613) + 1,000 µl Stain Buffer (BD Pharmingen™ Stain Buffer (FBS) 500 ML RUO, BD Biosciences, #554656)] and incubating at room temperature in the dark for 5 minutes. Then 300 µl Stain Buffer (FBS) was added to each sample, and the stained cells were analyzed by ID7000™ Spectral Cell Analyzer (Sony Technology). The targeting antibodies are listed in Supplementary Table 11.

### Statistical analysis

The statistical analysis was done using the Student's *t*-test. All analyses were performed using the GraphPad Prism 5 Software. In CCK-8 assays, the IC_50_ values were calculated using the GraphPad Prism 5 Software. In the assays of spheroids and MCTSs, ANOVA was used to perform statistical analysis among the three groups of LLC/2, and then the Bonferroni correction was used as a post hoc test to examine significant differences between the groups. The cell proliferation curves were generated automatically by GraphPad Prism 5 Software based on these IC_50_ values. *P* <0.05 was considered statistically significant. All *P* values were two-tailed. No blinding or randomization was used. No samples were excluded from analysis. All criteria were pre-established. No statistical method was used to predetermine sample size and the sample size for all experiments was not chosen with consideration of adequate power to detect a pre-specified effect size. Variations were compatible between groups. *In vitro* experiments, such as qPCR, cell proliferation assays, and MCTS/spheroid assays, were routinely repeated three times unless indicated otherwise in Figure legends or main text.

## Results

### Combined mutation cbTP53 does not apparently change the conformation of TP53, but affects the protein-DNA interactions

To investigate the molecular details of three selected hotpot residues —Y220, R248, and R282 —and the effects of their mutants (Y220C, R248Q, and R282Q), we analyzed the available crystal structures of TP53 and its complex with DNA. The wild-type TP53-DNA structures were aligned based on a single p53 monomer from the assembly (**Fig. 1A**). The RMSD values for all Cα atoms ranged from 0.5 to 1 Å, indicating a high degree of structural similarity between the overall structures. Y220 is a crucial component of the hydrophobic core of the β-sandwich structure and is located far from the promoter DNA. R248 interacts with the DNA's phosphate backbone and contributes to the stabilization of the complex. R282 plays a role in stabilizing the DNA-binding Loop-Sheet-Helix motif but does not directly contact the DNA (PDBs:1UOL, 1TUP) 16, 17.

Crystal structures of the targeted mutants, Y220C (PDBs:2J1X, 6SHZ, 8A32) 4, 18, 19 and R282Q (PDB:2PCX) 20, have been determined previously. However, no structure is available for the R248Q mutation. Consequently, we modeled this mutation based on the wild-type TP53-DNA structure (PDB:2AC0) 21 using ChimeraX, a structural analysis and visualization tool. Upon comparing the wild-type TP53 with the mutants (**Fig. 1B**), we observed that the overall structures showed subtle differences, with each mutation introducing localized structural changes.

Specifically, Y220 and its aromatic ring are essential components of the hydrophobic core of the local β-sandwich structure. The Y220C mutation results in the loss of the aromatic ring, creating an extended cavity that exposes hydrophobic residues to the solvent (**Fig. 1B**). This hydrophobic core, composed of Y220 and the surrounding prolines (151, 222, and 223), with π-π-interactions among them, plays a critical role in maintaining the structural integrity of the protein (**Fig. 2 Ai and Aii**) 3. Previous studies have shown that the Y220C mutation is highly destabilizing, reducing the thermal stability of the protein by approximately 4 kcal/mol 22. This mutation is temperature dependent: DNA binding is not significantly affected at low temperatures (retaining up to 67% of the wild-type affinity at 20 °C) but is lost at 37 °C due to protein denaturation. The cavity created by the Y220C mutation is located far from the DNA-binding sites, and its hydrophobic nature makes it an attractive target for drug design (**Fig. 2 Aiii**). This mutation has been extensively studied and targeted with various compounds, such as the JC769 compound, to stabilize the TP53 structure 18, 23, 24.

The R282Q mutation affects the packing of the Loop-Sheet-Helix motif, which binds the major groove of DNA 18. This mutation has a notable impact on the loops and disrupts DNA binding. R282 forms hydrogen bonds with T118, T125, Y126, S127 and E286 (**Fig. 2 Bi**). The crystal structure (PDB:2PCX) 20 reveals that interactions with T118, T125 and E286 are lost upon mutation, while Q282 forms a new interaction with the backbone of S127 instead of its side chain and with S116 (**Fig. 2 Bii**). Loop 1, which is initially flexible, became more rigid, leading to a shift in the position of K120 (**Fig. 2 Biii**). This shift compromises DNA binding due to a potential clash (**Fig. 2 Biv and Bv**), abolishing sequence-specific DNA contacts 21. Additionally, the rotation of H115 may affect overall protein folding 20.

The R248Q mutation was initially considered a DNA contact mutation, and extensive research has shown that it significantly reduces or abolishes DNA binding due to its role in contacting the minor groove of DNA. Recent NMR and MD simulation studies have also revealed two key effects: disruption of DNA contact and structural destabilization 25-28. No crystal structures are available for the R248Q mutation. Based on the wild-type TP53 structure (PDB:2AC0), R248 interacts with the phosphate backbone of DNA at T6 and A7 on one strand and G12 on the other (**Fig. 2 Ci**). Mutagenesis modeling in ChimeraX shows several potential orientations for the Q248 mutation (**Fig. 2 Cii**). Despite the mutation weakening DNA binding, it is not completely lost, as glutamine can still interact with the phosphate backbone. Functional studies confirm this, showing that while the mutation reduces mRNA levels, it does not completely abolish them 29.

Collectively, based on these structural analyses and comparisons of the available crystal structures, it is plausible that the proposed multiple mutations in an individual TP53 gene molecule does not apparently alter the overall conformation of the TP53 protein-DNA interactions directly. However, it may induce local conformational shifts within the neighboring regions or potentially destabilize protein under physiological temperatures, which could affect DNA binding affinity.

### Y220C and cbTP53 display divergent capacity to promote lung cancer growth *in vitro* and disease progression in mice

Multi-hit TP53 mutations imply an enhanced, non-additive tumorigenic potential. To dissect this, we focused on three mutation sites: Y220C, R248Q, and R282Q. R248 and R282 represent canonical "hotspot" codons within the DNA-binding domain (DBD) 2, whereas Y220C is situated outside this surface. While these individual residues are frequently mutated in non-small cell lung cancer (NSCLC)—with frequencies up to 10-15% for Y220C, and 30-50% for R248 and R282 variants—their coordination within a singular p53 molecule remains unexplored.

To isolate the functional synergy of the compound triple-mutant configuration (Y220C/ R248Q/R282Q, termed cbTP53), we generated lentiviral vectors encoding human single mutant (Y220C), DBD double mutant (R248Q/R282Q), and intermediates (R248Q/Y220C and R282Q/Y220C). LLC/2 cells were transduced and enriched via FACS to > 95% purity (Fig. S1A). Stable expression was confirmed by Western blot (**Fig. 3A; Fig. S1B**).

Real-time 2D proliferation kinetics via Incucyte revealed a striking epistatic interaction (**Fig. 3B**). The single mutant Y220C displayed the most rapid raw proliferation rate. However, a second mutation collapsed this advantage: the R248Q/R282Q control exhibited the slowest growth rate (dominant-negative effect), R282Q/Y220C showed blunted growth, and R248Q/Y220C proliferated identically to the Scramble control. Remarkably, the third mutation in cbTP53 partially rescued this deficit to yield an intermediate phenotype. This indicates a structural incompatibility where a second mutation disrupts the Y220C architecture (inducing instability or aggregation), whereas the third mutation acts as a structural compensatory event that restabilizes the molecule.

In 3D anchorage-independent environments, a divergent phenotypic hierarchy emerged. In Matrigel Dome assays, cbTP53 generated a significantly higher total number of clonal spheroids compared to Y220C, the R248Q/R282Q control, and both intermediate double mutants. Individual spheroid sizes remained comparable, indicating that cbTP53 specifically enhances sphere-initiating efficiency and survival rather than simple volumetric growth (**Fig. 3C** and** 3E; Fig. S1C** and** S1D**). Furthermore, in Multicellular Tumor Spheroid (MCTS) models, cbTP53 cells generated a significantly higher volume of unanchored aggregates but fewer compact MCTSs compared to Y220C and Scramble controls (**Fig. 3D**). While Y220C cells formed dense MCTSs—reflecting a restricted capability to adapt to detached matrix environments or a lower pool of cancer stem cells (CSCs)—the robust formation of non-adherent clusters by cbTP53 cells indicates enhanced cell-to-cell cohesion, anoikis resistance, and microenvironmental survival capacity. Collectively, these findings demonstrate that cbTP53 phenotypes do not arise from additive effects. Instead, they stem from a coordinated molecular evolution that shifts cellular dynamics away from rapid 2D division toward superior 3D anchorage-independent survival, immune evasion potential, and architectural fitness.

Because cancer patients with multi-hit *TP53* mutations tend to have significantly shorter median overall survival compared to those with single-hit mutations, we sought to test whether cbTP53 and Y220C own different tumorigenicity. Thus, 2 × 10^4^ LLC/2 cells expressing Y220C or cbTP53 were injected intravenously via the tail vein into syngeneic C57BL/6 mice, mimicking the natural process of tumor cells entering the bloodstream and circulating to the lungs. As shown in **Fig. 3F-3I**, cbTP53 cells have higher potential of cancer engraftment, in which 5 out of 9 (vs 1 out of 9 in Y220C) mice have heavier lungs with metastatic lesions and tumor nodules (*P* = 0.0221), consistent with the higher potential of forming more spheroids and aggregates. While liver is an organ that lung cancer cells usually metastasize to, we do not see metastatic lesions in liver, heart, spleen and kidney, which may take a longer time to develop metastatic cancer there. Although no obvious changes in the volume and weight of heart, liver, and kidney were seen, mice bearing cbTP53 cells than Y220C cells tend to have larger and heavier spleens (*P* < 0.001), an indicator of worse prognosis in lung cancer patients 30, 31. In agreement with these findings, H&E staining revealed that Y220C lungs contain scattered nodules with irregular, glandular, or lepidic growth preserving some alveolar architecture. In contrast, 3mut lungs exhibit solid masses that completely obliterate the parenchyma and alveolar spaces (**Fig. 3J**, left). In the spleen, Y220C displays distinct boundaries between a prominent, peripheral white pulp and a larger, lightly stained red pulp. Conversely, the 3mut group shows uniform deep staining and blurred boundaries caused by white pulp expansion (lymphocyte hyperplasia), which compresses the red pulp and suggests an enhanced immune response (**Fig. 3J**, right). No obvious differences were observed in the liver (**Fig. S2A**). IHC staining for BCL1 (Cyclin D1) revealed a trend toward lighter intensity in Y220C lungs compared to 3mut lungs, while Ki-67 expression showed no significant difference between the two. In the spleen, however, the Y220C group exhibited a downward trend in BCL1 levels alongside an increase in Ki-67 staining relative to the 3mut group. (**Fig. 3K; Fig. S3B-3E**). Collectively, these results suggest that lung cancer cells expressing 3mut (Y220C/R248Q/R282Q) possess higher tumorigenic and metastatic potential *in vivo*.

### Lung cancer cells carrying cbTP53 or Y220C mutations have dissimilar sensitivity to chemotherapeutic drugs and TP53 inhibitors

*TP53* mutations cause poor responses to various therapies 32, 33, including immunotherapy and chemotherapy. Importantly, multi-hit mutations lead to even worse prognosis and more resistance to therapy than single-hit (monoallelic) controls in various cancers. To this end, we examined the responses of LLC/2 cells containing Y220C or cbTP53 mutations to three most widely used drugs for lung cancer 34 - cisplatin, etoposide and carboplatin. We found that, compared to scramble, cells carrying Y220C or cbTP53 mutations have larger IC_50_ values (10.096 vs 16.9258 vs 8.0373 µM for carboplatin; 8.9876 vs 9.0671 vs 7.8753 µM for cisplatin; 12.3113 vs 20.3635 vs 10.2812 µM for etoposide; **Fig. 4A**). Changes of IC_50_ values for carboplatin and etoposide are more obvious than cisplatin. Treatment with 8 µM of carboplatin (IC_50_ value) impaired >80% potential of scramble cells to form spheroids; in contrast, ~40% or 45% of spheroid-forming potential remained in Y220C or cbTP53 cells, respectively (**Fig. 4B** and** 4C)**, indicating that cbTP53 cells own the highest resistant potential to carboplatin killing. These findings validate the concept that multi-hit TP53 mutations render cancer cells more therapy resistance, leading to poorer outcomes in lung cancer.

Mutant TP53 can lead to drug resistance through DNA damage and impaired DNA repair. To test the role of cbTP53 in DNA damage, we treated LLC/2 cells expressing scramble, Y220C, or cbTP53 with 8 µM carboplatin (IC_50_ value for scramble control), because carboplatin binds to and damages DNA preventing it from being repaired or copied. The comparative comet analysis revealed patterns of comets for Y220C and cbTP53 in contrast to scrambled controls (**Fig. 4D**). We calculated the changes in cells with a comet tail/tail DNA among three groups without considering tails' length and width. As shown in **Fig. 4E**, cells with tail DNA are rarely detectable and three groups have similar patterns of comet cells before treatment. Upon exposure to carboplatin 24 or 48 hours, no changes in comet cells were seen in cbTP53 cells, implying its resistance to carboplatin-initiated DNA damage or/and enhanced capacity to DNA repair; in contract, 3-4 folds or 1-2 folds increases of comet cells were seen in control or Y220C cells (*P* < 0.05), respectively, suggesting that both Y220C and cbTP53 are relatively sensitive to carboplatin-induced DNA damage, which warrants further investigation. Because the tail moment has been suggested to be an appropriate index of induced DNA damage in considering both the migration of the genetic material as well as the relative amount of DNA in the tail, we measured tail moment length from the center of the head to the center of the tail. We found that LLC/2 cells carrying Y220C or cbTP53 have significantly larger Olive Tail Moment 48 hours post treatment (**Fig. 4F**). Notably, we just quantified comet cells, a cell that contains a tail and a head like a comet; but not apoptotic cell, a cell with a large tail and a small head. This data implies that multiple cbTP53 mutations render lung cancer cells more advantages to resist DNA damage or facilitate DNA repair when compared to single mutations.

Given the pathogenic role of *TP53* mutations in cancer pathogenesis and drug resistance, TP53 mutations are attractive therapeutic targets 35, 36. We selected inhibitory molecules MP710 and PK11007, because PK11007 stabilizes p53-Y220C protein via selective alkylation of two surface-exposed cysteines, but MB710 binds tightly to the Y220C pocket and stabilizes p53-Y220C. CCK-8 assays revealed that cbTP53 (Y220C+R248Q+R282R) and Y220C cells show differential sensitivity to MP710 and PK11007 with the lowest for cbTP53 cells (**Fig. 4G**), implying that the induction of R248Q+R282R mutations in cbTP53 leads to a “subtle” change in the binding affinity of MP710 or PK11007 to Y220C, likely due to the local conformational shifts (refer to Fig. 1 and 2), which requires further investigations.

### Y220C and cbTP53 divergently rewires transcriptomic profiles in lung cancer cells

To investigate the mechanisms underlying the cellular response to combined TP53 mutations, we performed unbiased transcriptome-wide RNA-seq in LLC/2 cells expressing scramble (SC), Y220C or cbTP53. Both principal component analysis (PCA) and heatmaps revealed high concordance within triplicates and clear separation of gene expression among these cells (**Fig. 5A**,** 5B**; **Table S1**). Heatmaps (**Fig. 5C**; **Fig. S3A** and** S3B**) and volcano plots (**Fig. 5D**; **Fig. S3C** and **S3D**) were further utilized to visualize the differentially expressed genes (DEGs) in various comparisons. Among them, the upregulated genes are 1,584, 1,521 or 1,004, but downregulated genes 1,091, 1,681 or 1,747, in Y220C vs SC, cbTP53 vs SC or cbTB53 vs Y220C, respectively (FC <1.5 or >-1.5). Thus, the observed widespread cellular responses to *TP53* mutations (cbTP53) are likely mediated by transcriptomic changes.

To identify the biological pathways influenced by the observed transcriptome changes, we conducted Gene Ontology (GO) term enrichment analysis on the sets of up- and down-regulated genes. Compared to SC group, both Y220C (**Fig. S3E** and** S3F**; **Table S2**) and cbTP53 (**Fig. S3G** and** S3H**; **Table S3**) mutations showed significant enrichment in processes such as positive and negative regulation of polymerase II activity, positive regulation of gene expression, cell differentiation, regulation of cell population proliferation, and immune response. These findings were further corroborated by analyzing cbTP53 mutations in comparison to Y220C (**Fig. 5E**; **Fig. S3I**; **Table S4**), collectively reinforcing the idea that *P53* mutations play a crucial role in gene expression regulation and the control of cancer cell growth. Moreover, KEGG analysis revealed that pathways such as cytokine-cytokine receptor interaction, pathways in cancer, immune responses, and neuroactive ligand-receptor interaction were significantly enriched in Y220C (**Fig. S4A** and** S4B; Table S5**) or cbTP53 (**Fig. S4C** and** S4D**; **Table S6**) compared to SC. Further evidence for these pathway alterations was provided by KEGG analysis comparing cbTP53 and Y220C cells (**Fig. 5F**; **Fig. S4E**; **Table S7**). GSEA analysis of DEGs in Y220C vs SC, cbTB53 vs SC or cbTB53 vs Y220C comparisons identified common hallmark pathways, including inflammatory response, unfolded protein response, TP53 pathway and interferon gamma pathways (**Fig. 5G** (left); **Fig. S5A-S5D**). Notably, the Hallmark TNFA signaling via NFκB appears in cbTB53 vs SC or cbTB53 vs Y220C, but not in Y220C vs SC (**Fig. 5G** (right); **Fig. S6A** and** S6B**). These findings suggest that R248Q and R282Q in cbTP53 significantly alters the regulatory landscape observed in Y220C. This is further supported by the identification of 1,533 overlapping DEGs through Venn diagram analysis (**Fig. 5H**; **Table S8**), and the findings that 40-50% of genes differentially expressed between cbTB53 and Y220C were distinct, indicating altered regulatory properties in Y220C due to the R248Q and R282Q mutations. Overall, these results demonstrate that cbTP53 substantially modifies the transcriptomic profile of lung cancer cells, leading to observable changes in cellular behaviors.

### cbTP53 and Y220C differentially regulate cytokine expression through divergent binding affinity in the promoters of target cytokines in lung cancer cells

Lung cancer progression partially results from the communications of tumor cells with host microenvironment. Such communications occur through the release of cytokines, chemokines and growth factors from cancer cells 37. Further, TP53 is an essential regulator for cytokine expression in tumor cells 38-40, actively reshaping the profile of cytokines and chemokines secreted by cancer cells *in vitro* and *in vivo*. To explore the impacts of cbTP53 mutations on cytokines in cancer cells, we subjected the culture medium from LLC/2 cells with scramble, Y220C or cbTP53 to cytokine arrays (Mouse Cytokine Array Panel A; 40 mouse cytokines). We found that, compared to control and Y220C, cbTP53 induces the most pronounced upregulation of CXCL1/2, TNF-α, MCSF, INF-γ and TIMP1 with no obvious changes in CCL2 release (**Fig. 6A** and **6B**). qPCR verified the upregulation of CXCL2, TNF-α, INF-γ and TIMP1, although expression of CCL2 and CXCL1 is decreased with no obvious changes of MCSF in cbTP53 vs Y220C (**Fig. 6C**). Given the upregulation of released cytokines in cbTP53 culture medium, the more production of cytokines supports the more contribution of cbTP53 to pro-tumorigenic microenvironment, substantiating the observations that the upregulation of TIMP1, CSF1 and TNF tends to predict unfavorable outcomes in patients with lung adenocarcinoma (**Fig. S7**).

To elucidate the biological functions and pathways impacted by the 6 altered cytokines, GO and KEGG enrichment analyses were performed. The network of significantly enriched KEGG pathways were displayed in **Fig. 6D**, which highlighted NFκB signaling, TNF signaling, and cytokine-cytokine receptor interaction as key direct pathways associated with these cytokines. GO analysis revealed that these 6 cytokines significantly influence 17 out of 27 identified cellular functions (FDR ≤ 0.05), including NFκB and TNF signaling, as presented in **Fig. 6E**. The critical role of NFκB and TNF signaling pathways in cbTP53-driven inflammation was further substantiated by bulk RNA-sequencing data (ref. Fig. 5G; Fig. S5), demonstrating that TNFA signaling via NFκB is preferentially activated in cbTP53 vs. SC or Y220C, but not in the Y220C vs. SC comparison. Given the well-established importance of NFκB and TNF signaling in shaping the tumor microenvironment (TME) and their influence on lung cancer metastasis, these findings collectively suggest that multiple mutations may disrupt tumor cell-TME interactions, thereby promoting lung cancer progression.

TP53 proteins bind as homo-tetramers to the promoter regions of ~500 direct target genes that regulate apoptosis, cell cycle arrest, DNA repair and other cellular processes 41, 42. To dissect the mechanisms by which TP53 mutations enhance cytokine production, and to examine whether induction of more mutations in individual TP53 gene molecule alters DNA binding affinity, we performed ChIP assays in scramble, Y220C or cbTP53 LLC/2 cells. To reduce the impacts of endogenous mouse P53 protein on ChIP, we employed a P53 antibody that detects only human, but not mouse, P53 protein (ref. Fig. 3A). The PCR primers for quantifying ChIP immunoprecipitated complexes are located within 2 kb upstream of target promoters bearing potential P53 binding sites. While the regulatory patterns of cytokine expression show variations (up-, down- or no change; ref. Fig. 6B and 6C), the DNA binding affinity on all target cytokines tend to be higher in cbTP53 than Y220C cells (**Fig. 6F**). On the one hand, these findings suggest that multiple mutations on a single TP53 gene molecule could enhance P53 DNA binding activity, in line with the conformational shifts; on the other hand, other regulatory factors (e.g., protein interactions) are required to coordinate with TP53 mutations to determine ultimately the transcriptional outcomes, either up- or down-regulation of target genes.

### Circulating cbTP53 and Y220C lung cancer cells stimulate distinct immunological responses

TP53 mutations promote cancer development at least partially through perturbating immune cell functions. To this end, we injected C57BL/6 mice with LLC/2 carrying cbTP53 or Y220C mutations intravenously via the tail vein, mimicking the metastasis of circulating tumor cells to form lesions and tumor modules in lungs. Then we collected the whole blood (n = 5 mice/group, pooled) in 3 weeks after LLC/2 cell engraftment and conducted a comprehensive analysis of the overall immunologic state of cancer-bearing mice by staining the whole blood cells (after red blood cell lysis) to capture the full breadth of immunological perturbations or activation (**Fig. 7**). In Y220C vs cbTP53, 83.27 vs 91.41% (live CD45^+^ cells), 66.02% vs 71.93% (myeloid cells; CD3e^+^/CD11b^+^), 44.33% vs 59.17% (B cells; CD3e^+^/B220^+^), 30.97% vs 25.77% (T cells; CD3e^+^/B220^+^), 5.9% vs 3.83% (NK cells; CD3e^+^/NK1.1^+^), 45.34% vs 40.15% (CD8^+^ T; CD4^-^/CD8^+^), 47.72% vs 53.12% (CD4^+^ T; CD4^+^/CD8^-^), 73.51% vs 64.5% (Treg; CD25^+^/CD127^-^), 29.73 vs 34.97% (Monocyte; CCR2^+^/LyC6^+^) as well as 0.07% vs 0.41% (circulating tumor cells, CTCs; CD45^-^/PD-L1^+^). Notably, lower number of T cells, NK cells and CD8^+^ T cells are frequently associated with worse prognosis and tumor aggressiveness, with a contradictory observation of lower treg cells; In contract, high levels of myeloid cells and monocytes usually predict the enhanced lung cancer disease, whereas high counts of B cells and CD4^+^ T cells have a paradoxical role in the context of increased lung cancer aggressiveness and a poorer prognosis. Further, mice bearing cbTP53 have higher levels of circulating CD45^+^/PD-L1^+^ cells (white blood cells expressing both CD45 and PD-L1) than those bearing Y220C (91.6% vs 83.46%; H section), which is a reliable predictive biomarker linking to increased aggressiveness in lung cancer. Importantly, mice bearing cbTP53 cells have a much higher number of CTCs than Y220C cells (0.07% vs 0.41%). Given that higher CTCs are strongly associated with increased aggressiveness and poorer prognosis in lung cancer, these findings suggest that cbTP53 cells establish a more immunosuppressive TME in mice, and have larger capacity to survive and proliferate *in vivo*, leading to worse metastatic diseases (refer to Fig. 3F-3I).

## Discussion

It is well documented that, compared to single-hit (SH) mutations, multi-hit (MH) TP53 mutations are associated with worse prognosis and poorer outcomes in cancers compared to single-hit (SH) mutations 14, 43 with largely unknown causes. It is also well known that multiple TP53 mutations are frequently detected in a single cancer cell, a single tumor or within a single tissue 44, challenging the long-held notion that a single TP53 mutation is sufficient to initiate and promote tumorigenesis. Two critical questions remain unanswered: 1) whether multiple TP53 mutations can occur within a single TP53 gene molecule (cbTP53), and 2) if so, whether and how cbTP53 mutations might alter the nature of a TP53 gene molecule. In this study, we exploited the paradigm of cbTP53 effects on the structural, transcriptional and oncogenic properties of TP53 gene. We present a comprehensive dataset documenting significant changes in protein conformation, DNA binding affinity, transcriptional regulation, drug resistance, and tumorigenic potential due to combined cbTP53 mutations. Our mechanistic findings suggest that, compared to the Y220C mutation, cbTP53 differentially upregulates inflammatory cytokines, thereby promoting aggressive lung tumorigenesis through enhanced DNA binding affinity at the promoters of target cytokines.

While it is uncertain whether and how frequently cbTP53 exists, particularly in cells from cancer patients, as proof of concept, we engineered a vector expressing Y220C+R248Q+R282Q in a single TP53 gene molecule. This is because that R248Q and R282Q are recognized as two of the six major hotspot mutations located within the DNA-binding domain of TP53 2. In contrast, Y220C represents the most prevalent cancerous mutation found outside the TP53 DNA-binding surface 3. It's important to note that these hotspot mutations, including Y220C, constitute up to 25% of all documented p53 mutations 4. Our initial structural analyses, based on existing crystal structures, did not reveal remarkable changes in the overall conformation of cbTP53. Nevertheless, our findings suggest that cbTP53 may influence protein-DNA interaction affinity by modulating local stability or thermal stability. This was evidenced by our ChIP assay, showing that cbTP53 exhibits increased binding to the promoters of all tested cytokines, though this binding did not correlate with cytokine expression levels. However, the limitation is that the predicted changes in structural conformations of cbTP53 protein fully rely on computer modeling, but not crystal of TP53 protein carrying such mutations. Because mutations in R248 dramatically change TP53 structure impairing the formation of protein crystal, the validation of our computation predictions by TP53 protein crystal may be challenging. Moreover, potential changes in thermal stability and methodological specificity may limit the clarity of insights gained from crystal structures.

TP53, a critical transcriptional factor, activates or represses target genes through direct or/and indirect promoter DNA binding. WT TP53 generally exhibits a high affinity for specific DNA sequences, crucial for its tumor suppressor activity, whereas mutations within DBD reduce or eliminate such DNA-binding ability in the promoters of certain genes 45. Interestingly, single TP53 mutations can also upregulate target genes (e.g., CXCL1, MDR1) by increasing enhancer activity or through cofactor-mediated promoter binding 39. However, the DNA binding affinity of combined mutations (cbTP53) compared to single mutations (e.g., Y220C) remains unknown. Our structural analyses revealed local conformational shifts in cbTP53 compared to Y220C or WT TP53, despite the lack of apparent changes in overall confirmation. This alteration could ultimately affect DNA binding affinity. Indeed, ChIP assays demonstrated that cbTP53 exhibits stronger binding to the promoters of cbTP53-targeted cytokines (e.g., CXCL2, TNFα and IFN-γ) compared to both the Y220C single mutation and a negative control. Whether this enhanced binding is a direct cbTP53-promoter interaction or mediated by physical interactions with TP53 protein partners requires further investigation. Furthermore, this conclusion, drawn from a limited set of upregulated cytokines, necessitates broader validation through techniques like lectrophoretic mobility shift assay (EMSA) or ChIP sequencing. Given that TP53 transcriptional regulation is significantly influenced by DNA binding, further studies on the interactions between TP53 and its partner proteins is necessary to substantiate our findings that cbTP53 mutations lead to enhanced transcriptional regulatory functions, particularly in light of the observed local conformational changes in cbTP53 which could interfere with its ability to interact with partner proteins.

Cancer cells with multi-hit TP53 mutations consistently demonstrate a diminished response to chemotherapy compared to those with single-hit TP53 mutations. The most frequently cited mechanism suggests a correlation between multi-hit TP53 mutations and more complex genetic abnormalities. However, it remains unclear whether and how combined multiple mutations within a single TP53 gene molecule (cbTP53) itself directly contributes to this reduced drug response. This project structurally and experimentally investigated this issue, demonstrating that cbTP53 cells differently respond to chemodrugs such as carboplatin, cisplatin and etoposide when compared to cells with the Y220C mutation. This differential response may stem from their distinct rates of cell proliferation, spheroid formation, and MCTS/aggregate development. Furthermore, cbTP53 cells exhibit varying sensitivity to Y220C inhibitors like MP710 and PK11007, whose inhibitory activity relies on drug binding to TP53 protein. This reduced sensitivity could be attributed to conformational changes in cbTP53 protein, which may affect the binding affinity of MP710 or PK11007, warranting further investigation. These compelling findings provide a plausible explanation for the observed limited effectiveness of MP710 and PK11007 in patients, suggesting that selective clonal expansion of cells harboring combined mutations like cbTP53 may enable resistance to these targeted therapies. A key limitation here is the subtle difference in chemodrug and Y220C inhibitor responses between Y220C and cbTP53. Nevertheless, given that development of drug resistance in heterogenous cancer is a process of clonal selection and evolution under drug pressure, such “subtle” differences in a few resistant/tolerant clones are profoundly significant. They are sufficient to increase cell plasticity, allowing clones to escape initial drug-induced killing, gain a competitive advantage over sensitive cells, and eventually expand into drug-resistant populations.

A critical mechanism driving TP53-mutation-associated cancers involve disrupted tumor microenvironment (TME) communication driven by cytokine secretion from cancer cells 47. Consistent with this, LLC/2 cells expressing cbTP53 form more spheroids and aggregates *in vitro*, and produce more aggressive lung disease in mice. Mechanistically, cbTP53 cells secrete higher levels of inflammatory cytokines (CXCL1, IFN-γ, TIMP1), whose elevation associates with shorter patient survival and skew host immune profiles toward immunosuppression. It requires further demonstration that a coordination between the secreted cytokines from LCC/2 cells and the reduction of “good” immune cells in cancer mice, which may be caused by LLC/2-secreted cytokines, creates a tumor-promoting TME leading to higher lung cancer burden. Limitations include: 1) cytokine arrays have limited sensitivity and detected only a subset of changes *in vitro*; more comprehensive proteomic arrays and RNA/protein sequencing in cell culture and serum from mice bearing cbTP53 LLC/2 cells are needed to validate cytokine regulation. 2) we overexpressed human TP53 mutants in syngeneic LLC/2 cells, so competition or interference from endogenous mouse p53 may contribute background signal; validation in human models and p53-null systems is warranted given TP53's prevalence in >50% of cancers. 3) the impact of our findings is centered on basic research field at this stage. Clinical relevance is uncertain because multiple mutations within a single TP53 allele have not yet been reported in patients.

Finally, while our study establishes cbTP53 (Y220C+R248Q+R282Q) as a potent driver of aggressive *in vivo* tumor burden, and enhanced microenvironmental fitness, certain limitations warrant consideration. First, our transcriptomic and ChIP assays collectively demonstrate a multi-faceted mechanistic model involving both dominant-negative (DN) suppression of the canonical p53 network and prominent neomorphic gain-of-function (GOF) behavior. This GOF phenotype operates via direct transcriptional hijacking of distinct mouse inflammatory cytokine promoters. However, the precise structural boundaries separating this co-existing DN effect from ectopic inflammatory reprogramming remain to be fully mapped. Second, because the murine lung cancer cell line LLC/2 retains wild-type endogenous p53, the potential formation of human/mouse p53 hetero-tetramers introduces biological complexity. Although we deployed a highly specific human-p53-selective antibody in our ChIP assays to confirm that exogenous human mutant proteins physically occupy endogenous mouse promoters, this native background complicates isolated data interpretation. Fully isolating the pure mutant-only phenotype from native p53 interference requires a clean, p53-null murine genetic background. Future work utilizing CRISPR-engineered p53-null LLC/2 lines or validations in p53-null bone marrow-derived systems will definitively rule out native tetrameric interference and resolve the exact kinetic trade-offs of this variant. Crucially, these constraints do not diminish our primary innovation: cbTP53 trades pure 2D kinetic speed for potent microenvironmental fitness, acting as an inflammatory reprogrammer rather than a simple proliferation driver to dictate tumor progression. These limitations do not undermine the central finding: cbTP53 shifts cells away from pure 2D proliferative speed toward enhanced microenvironmental fitness and inflammatory reprogramming that promotes progression. This conclusion is supported by i) structural evidence of local conformational shifts, ii) transcriptomic and ChIP data showing altered global gene expression and promoter occupancy of cytokines, and iii) functional assays demonstrating increased spheroid formation, reduced chemosensitivity, establishment of an immunosuppressive TME, and greater tumor burden *in vivo*.

## Conclusion

Compound TP53 mutations (Y220C/R248Q/R282Q) produce non-additive, epistatic effects: local structural shifts alter DNA-binding and transcriptomes, amplify pro-tumorigenic cytokine and NFκB/TNF signaling, enhance anchorage-independent growth and chemoresistance, and remodel systemic immunity to promote metastasis. Mechanistically, R248Q/R282Q perturb Y220C's local architecture to restore stability while changing promoter occupancy, explaining the divergent cellular phenotypes. These data define a novel multi-hit TP53 mechanism driving lung cancer aggressiveness and nominate mutational-context-dependent strategies for targeted stabilization or immune-modulatory therapy.

## Supplementary Material

Supplementary figures and table 9.

Supplementary table 1.

Supplementary table 2.

Supplementary table 3.

Supplementary table 4.

Supplementary table 5.

Supplementary table 6.

Supplementary table 7.

Supplementary table 8.

## Figures and Tables

**Figure 1:**
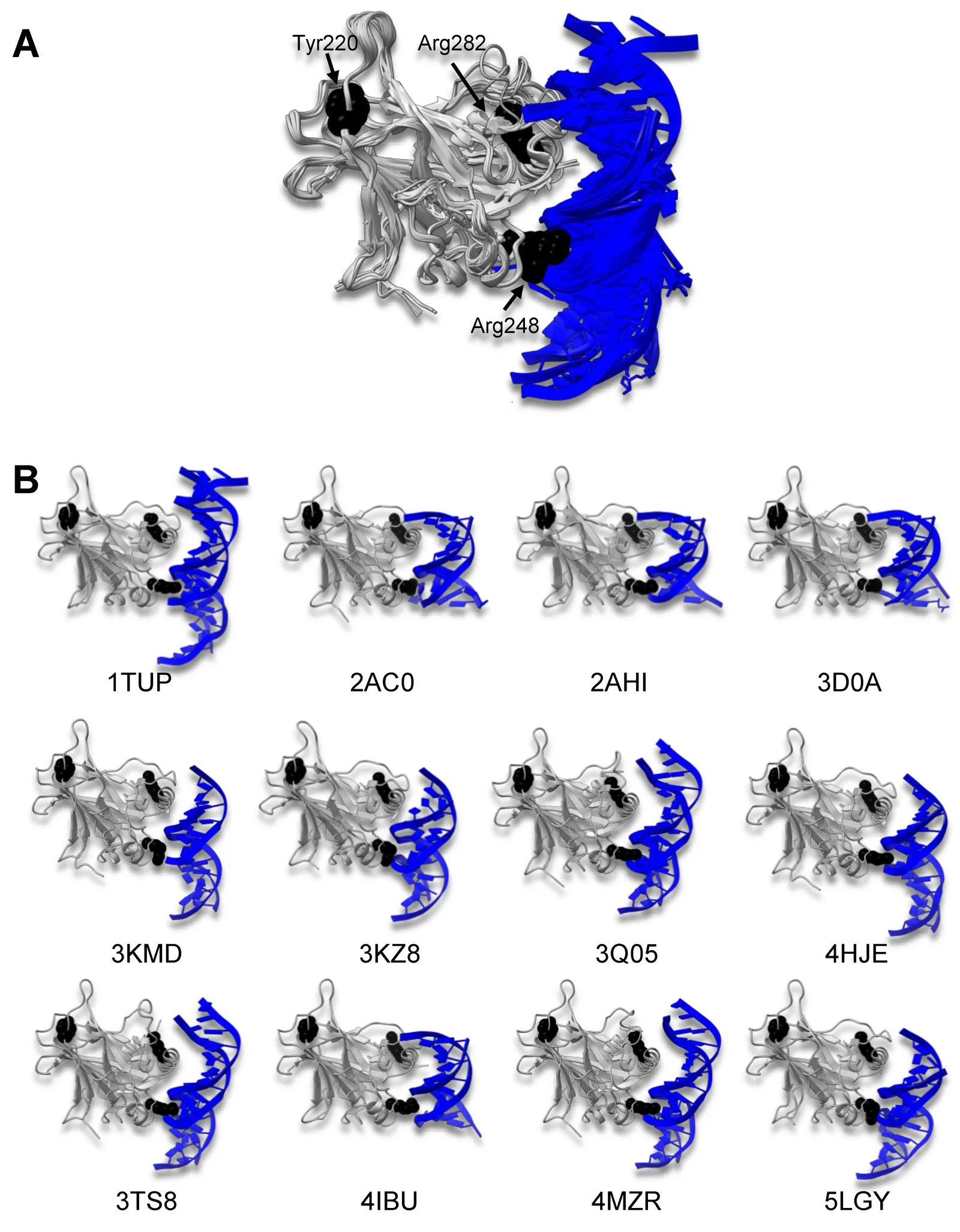
** Comparisons of available DNA-bound TP53 structures.** (A) Superposition of available TP53 structures based on the TP53 monomer. The alignment shows high structural identity among the wild-type TP53 bound to DNA and various mutant forms (3D0A: R249S and H168R, 4IBU: R273C and T284R, 4MZR: S121F and V122G). The three mutation sites studied in this research are highlighted as black spheres. The root means square deviation (RMSD) between the aligned structures ranges from 0.5 to 1 Å, indicating a very high degree of structural similarity across the different forms. (B) Individual structures of TP53/DNA complexes, each annotated with the corresponding PDB codes.

**Figure 2:**
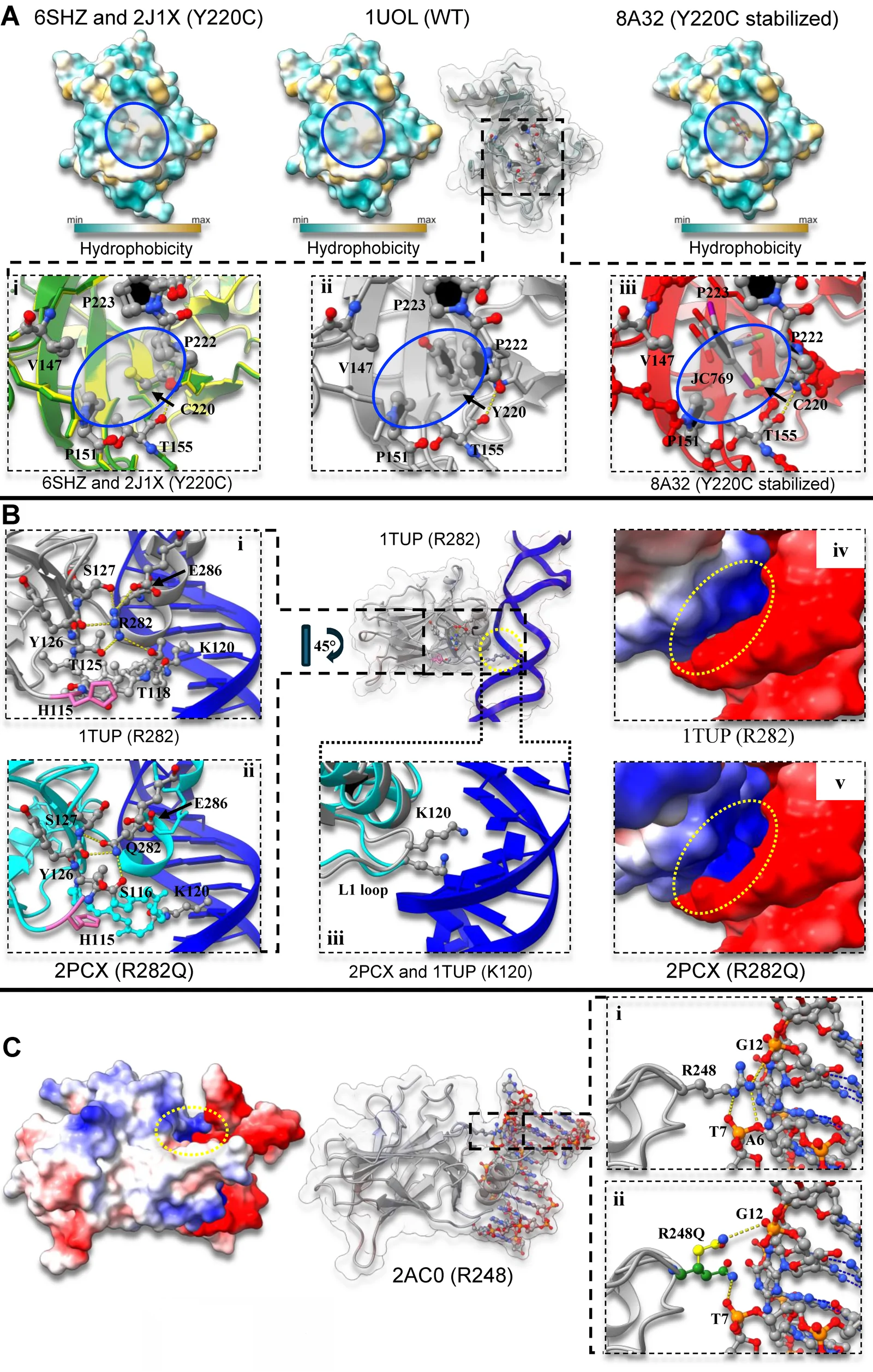
** Effect of studied mutations on TP53 structure.** (A) Y220C mutation. Interactions between amino acids are depicted with yellow dashed lines. The top panel shows an overall view of wild-type TP53 (gray, PDB: 1UOL) alongside the hydrophobic surface representation, compared with three mutant structures (PDBs: 2J1X in yellow, 6SHZ in green, and 8A32 in red). (i) Details of the Y220C mutant interaction with its environment. (ii) Detailed interactions of wild-type Y220 with its surrounding environment. (iii) Interaction of the Y220C mutant with its environment, stabilized by the JC769 compound. (B) R282Q mutation. Amino acid interactions are shown with yellow dashed lines. The middle panel presents the overall structure of wild-type TP53 (PDB: 1TUP). (i) and (ii) Show the detailed interactions between wild-type R282 (gray, PDB: 1TUP) and mutant Q282 (cyan, PDB: 2PCX) with their surrounding amino acids, with H115 colored in pink. (iii) A shift in the position of the K120 side chain. (iv) Display of the electrostatic surface at the K120/DNA interaction region in wild-type TP53-DNA (PDB: 1TUP). (v) Superimposition of the wild-type TP53-DNA and TP53/R282Q-DNA models suggests that the K120 side chain could potentially clash with the DNA backbone. (C) R248Q mutation. Protein-DNA interactions are represented by yellow dashed lines. The top panel shows the overall view of the PDB: 2AC0 structure and an electrostatic surface representation, with blue indicating positive charge and red indicating negative charge. (i) Depicts R248/DNA interaction, where the amino group of arginine interacts with the oxygen atoms of the DNA phosphate backbone. (ii) Illustrates potential orientations of the R248Q side chain in green and yellow, suggesting that DNA affinity could be significantly reduced but still present. The mutation was modeled using ChimeraX, with two rotamers of Q248 shown.

**Figure 3:**
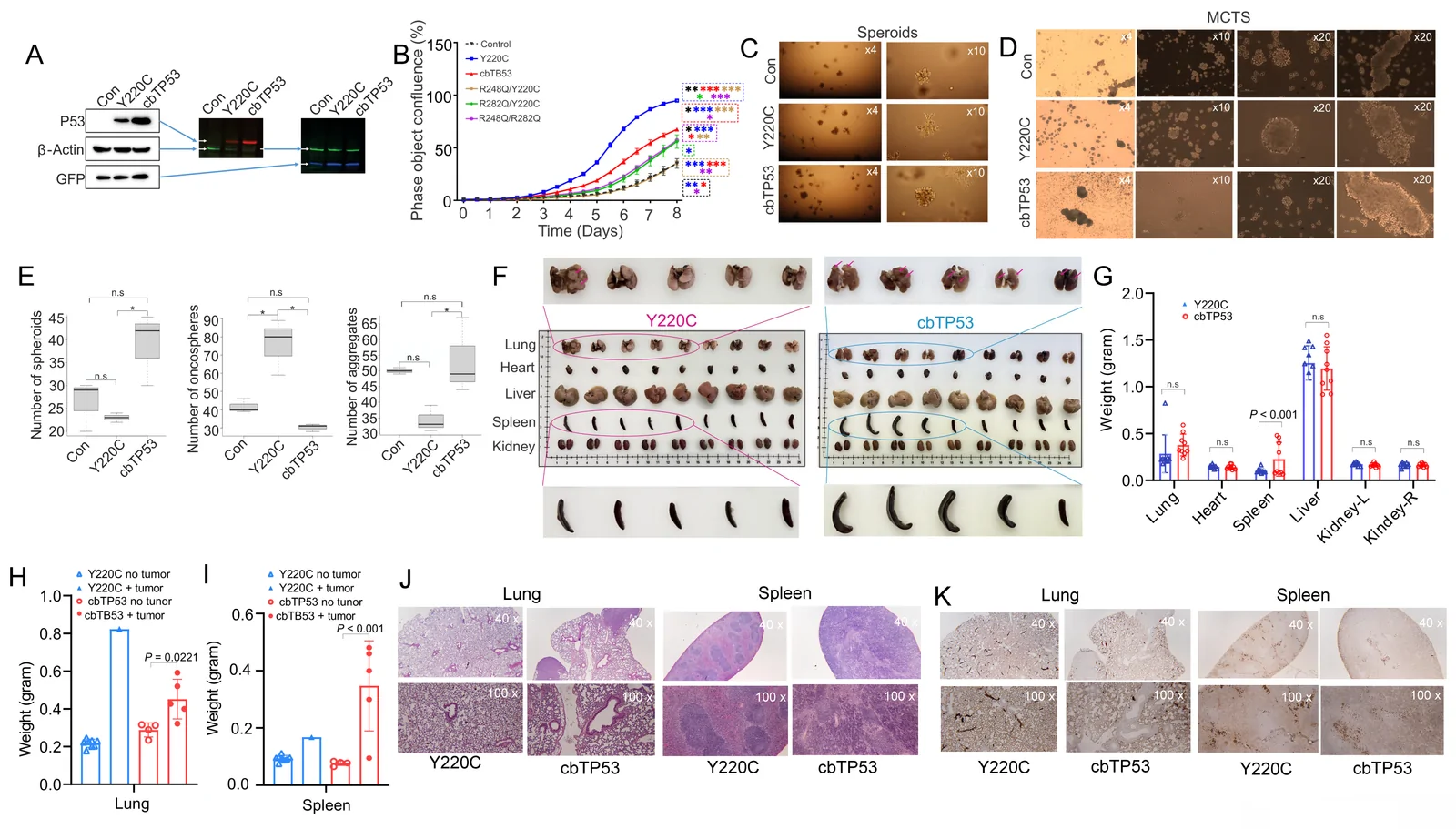
** cbTP53 mutations display distinctive phenotypic traits compared to Y220C and scramble control.** (A) Western blot is the expression of P53 and GFP proteins in LLC/2 cells overexpressing these genes, which was detected by 3 secondary antibodies. Red, P53 protein; Green, actin or GFP. Data represents 3 independent experiments. (B) IncuCyte live cell analysis monitoring proliferation of LLC/2 cells carrying different mutations. The kinetics of cell confluences calculated from raw data images. Data shown as the mean ± SD of triplicate experiments (P_Y220C **vs** control_ = 0.0042, P_cbTP53 **vs** control_ = 0.0112, P_cbTP53 **vs** Y220C_ = 0.0006, P_R248Q/Y220C **vs** control_ = 0.9371, P_R248Q/Y220C **vs** Y220C_ = 0.0003, P_R248Q/Y220C **vs** cbTP53_ = 0.0006, P_R282Q/Y220C **vs** control_ = 0.0626, P_R282Q/Y220C **vs** Y220C_ = 0.0229, P_R282Q/Y220C **vs** cbTP53_ = 0.2038, P_R282Q/Y220C **vs** R248Q/Y220C_ = 0.0707, P_R248Q/R282Q **vs** control_ = 0.0346, P_R248Q/R282Q **vs** Y220C_<0.0001, P_R248Q/R282Q **vs** cbTP53_ = 0.0127, P_R248Q/R282Q **vs** R248Q/Y220C_ = 0.0043, P_R248Q/R282Q **vs** R282Q/Y220C_ = 0.865). (C-E) 200 LLC/2 cells expressing scramble, Y220C or cbTP53 were subjected to spheroids- (C) and MCTS- (D) forming assays. Representative images of spheroids were taken on day 11, but MCTS and aggregates were taken on day 7. Graphs (E) are the quantification of spheroids, MCTSs or aggregates. Results are shown as mean ±SD. (F-I) About 2 × 10^4^ LLC/2 cells expressing Y220C or cbTP53 were injected into C57BL/6 mice (n = 9 mice/group) via the tail vein, and the cancer-bearing mice were sacrificed 3 weeks after initial engraftment. The middle images are external views of organs including lungs, heart, liver, spleen and kidneys; the upper images show representative visual analysis of lungs with/without metastatic lung tumors. Red arrows indicate the representative tumor nodules growing on lungs; the low panel images show the representative visual analysis of spleens; the graphs in G-I indicate the calculated weights of spleens, lungs, heart, liver, and kidneys. (J, K) Representative images of H&E (J) and IHC (Ki-67, K) staining of lungs and spleens reported in F (n = 3/group). Con, control/scramble; cbTP53, Y220C+R248Q+R282Q; ns, not statistically significant; MCTS, Multicellular Tumor Spheroids.

**Figure 4:**
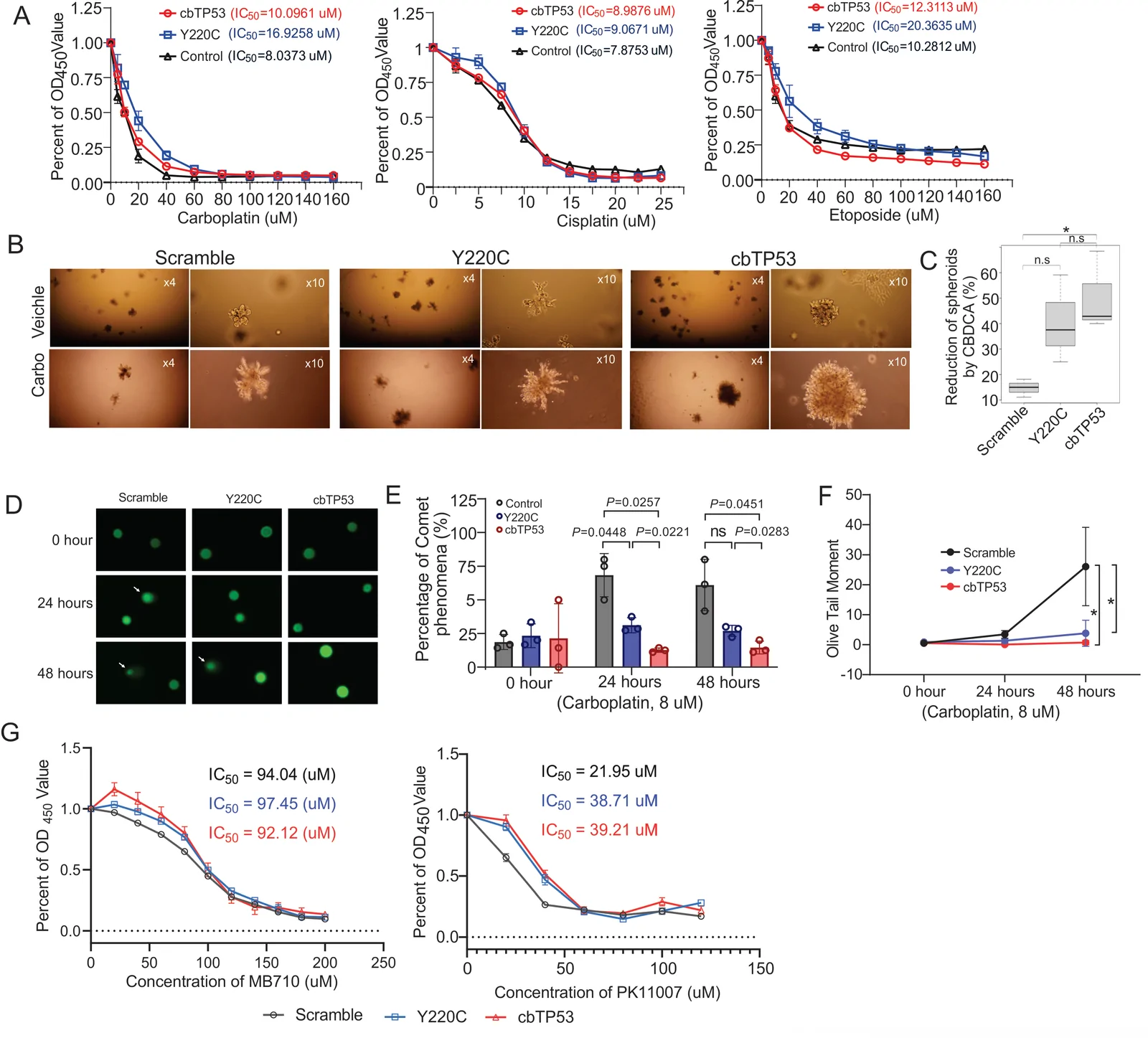
** The cbTP53 and Y220C mutations have different responses to therapeutic drugs.** (A) Results of CCK-8 assays in LLC/2 cells expressing scramble, Y220C or cbTP53 treated with various concentrations of carboplatin (48 hours), cisplatin (24 hours) or Etoposide (24 hours). The absorbance values were measured at 450 nm with a microplate reader. Data shown as mean values ±SD of n = 3 independent experiments done in quadruplicate. (B) About 200 LLC/2 cells expressing scramble, Y220C or cbTP53 were seeded in the gels for 24 hours and then carboplatin (48 hours), and then 8 µM of carboplatin was added. Representative images of spheroids were taken on day 11. (C) Graphs are the quantification of spheroid number (full gel area; triplicate; percentage). Results are shown as mean ± SD. (D-F) LLC/2 cells carrying different mutations were treated with 8 µm of carboplatin (IC_50_ values for 24 or 48 hours, and subjected to comet assays. Example images of DNA damage in (D) are three groups scramble, Y220C and cbTP53. Comet-patterns typical for cbTP53 show that chromosomal DNA is localized mainly to heads of comets (intact DNA). In contrast, images of scramble (24 and 48 hours) and Y220C (48 hours) demonstrated clearly damaged DNA (visible comet-tails and diffuse comet-heads). Graphs in (E) are percentages of comet phenomena for evaluation of DNA damage degree; Curves in (F) are the olive tail moment. cbTP53, Y220C+R248Q+R282Q; n.s, not statistically significant.

**Figure 5:**
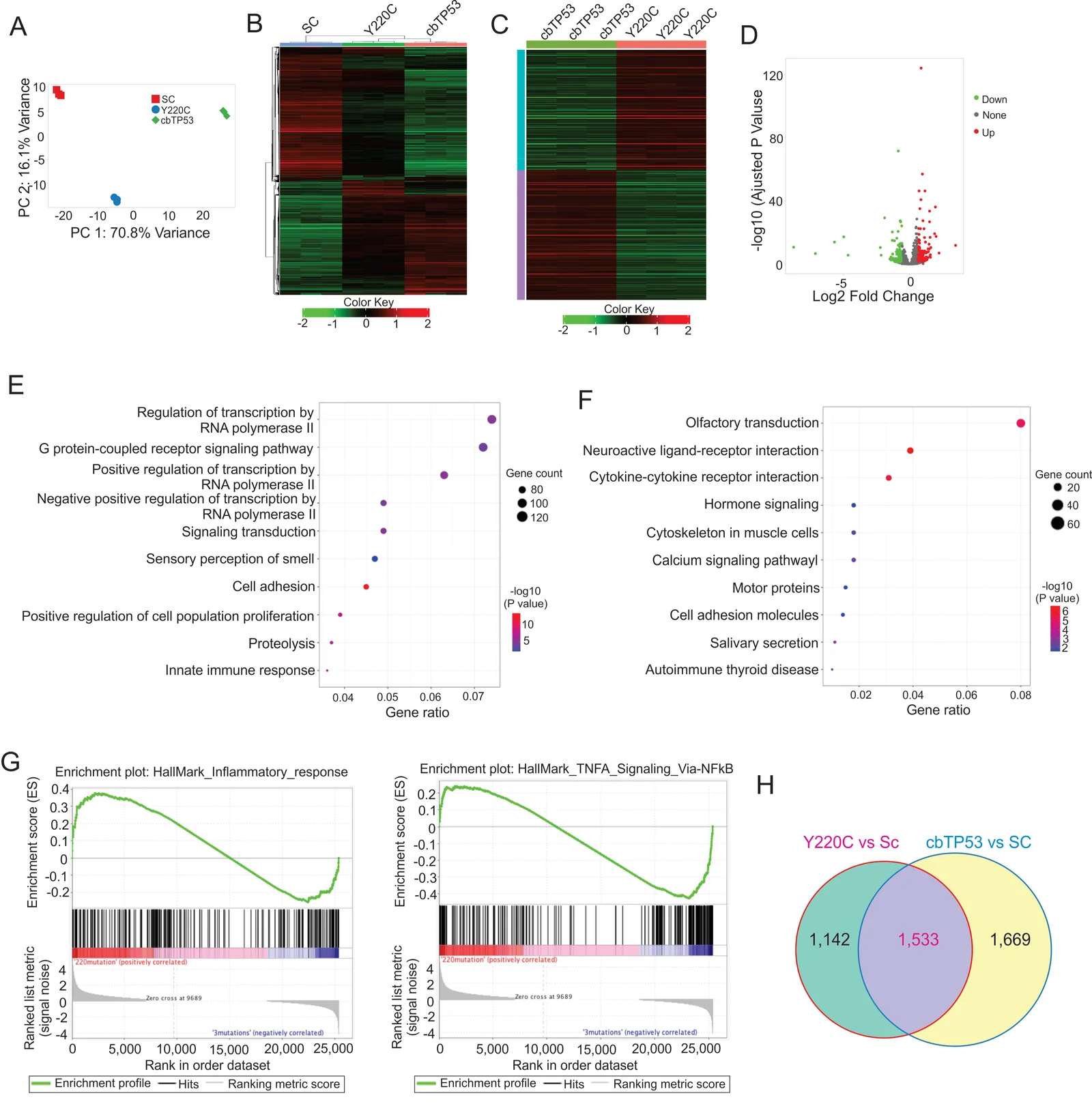
** Distinctive transcriptomic signatures are created by cbTP53 and Y220C in lung cancer cells.** (A) PCA score plots of gene expression data, illustrating the clusters along the PC1 and PC2 variance (%) by the treatment groups scramble (SC), Y220C and cbTP53. (B and C) Heat maps showing genes with differential expression (Log2 fold change ≥ 1.5 or < -1.5) among the triplicates of three groups (SC, Y220C and cbTP53 (B)) or in groups cbTP53 vs Y220C. Red and green indicate high and low expression, respectively. (D) Volcano plots showing the differentially expressed genes in groups cbTP53 vs Y220C. (E and F) Bubble plots showing the significantly enriched GO (E; top 10; *P* <0.05) and KEGG (F; top 10; *P* <0.05) pathways for upregulated genes in groups cbTP53 vs Y220C. Larger bubbles indicate a higher number of genes. Bubble color reflects significance (*P* value). (G) Gene set enrichment analysis of hallmark gene sets that are significantly enriched in groups cbTP53 vs Y220C. (H) A Venn diagram summarizing the overlap of differentially expressed genes (both up- and down-regulated) between groups Y220C and cbTP53 when compared to SC, respectively.

**Figure 6:**
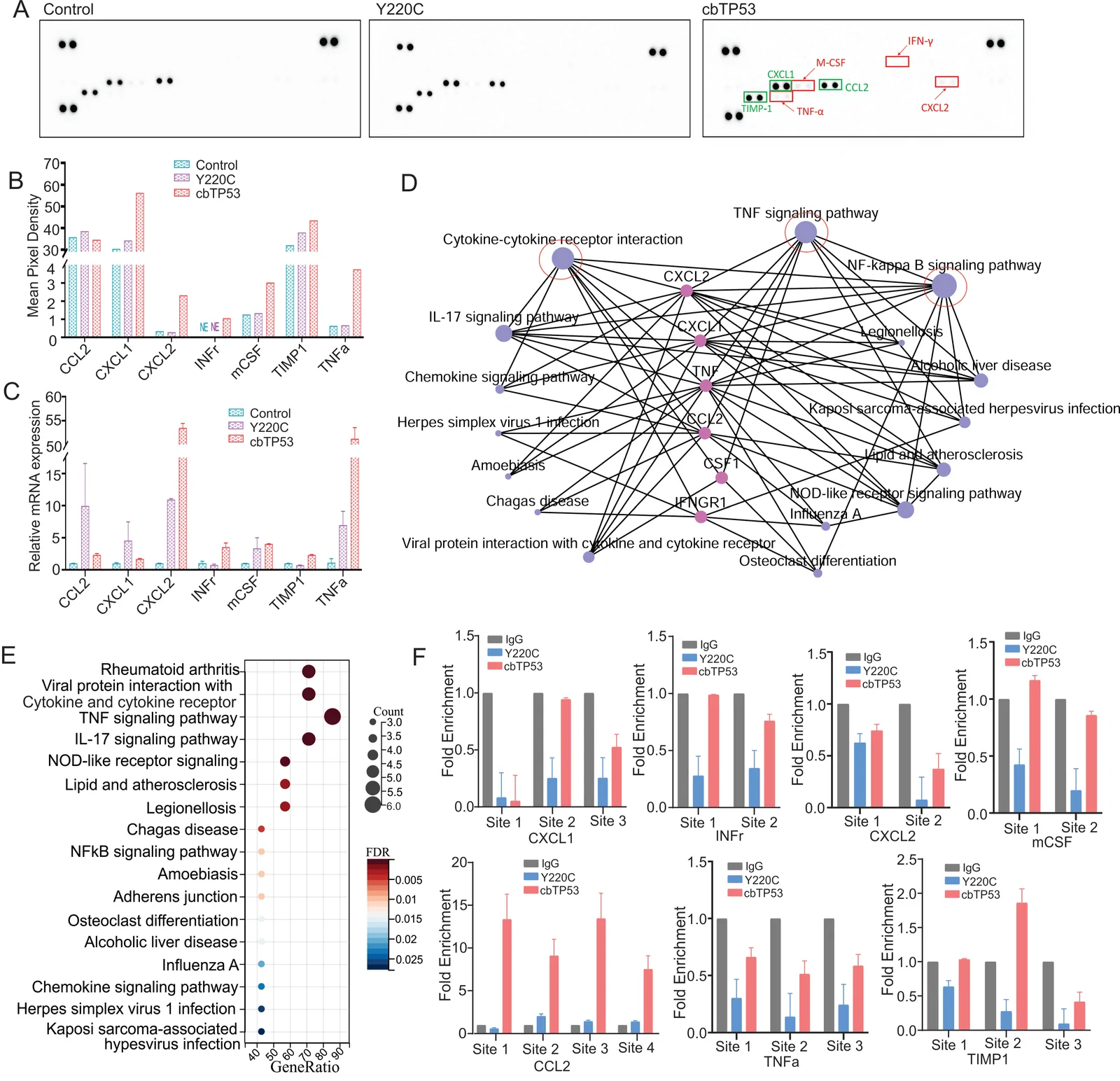
** The cbTP53 and Y220C have different cytokine expression profiles and DNA binding affinity in gene promoters.** (A) Cytokine antibody arrays incubated with cell culture from LLC/2 cells carrying scramble, Y220C or cbTP53 mutations growing in DMEM medium supplemented with exosome-depleted FBS for 72 hours (n = 2 samples/group). (B) Quantification of cytokines reported in (A) using image J. (C) qPCR measuring the differential expression of indicated cytokines in total RNA of LLC/2 cells expressing scramble, Y220C or cbTP53. Results represent 3 independent experiments. (D) The KEGG pathway-based network analysis of significantly changed cytokines in cbTP53-Y220C comparison (*P* <0.05). Red denotes gene nodes that are associated with cytokines identified by cytokine array, and light blue denotes gene nodes that were defined by KEGG in EV-AE compared to EV samples. The red circle (e.g., NFkB, TNF, Cytokine-cytokine receptor interaction) denotes the most shared node in comparisons. Larger circles represent larger enrichment scores. (E) Selected enriched Gene Ontology cellular/disease-enriched functions associated with up-regulated differentially expressed cytokines (DEGs). The top 17 by DEG counts are presented (FDR ≤0.05). The color of the dot depends on the value of FDR, and its size is determined by the number of DEGs related to the respective pathway in the analyzed set of DEGs (map color keys along with dot size ones are shown on the right). (F) ChIP assays examining the binding of Y220C or cbTP53 in the different regions of indicated cytokine promoters. The immunoprecipitated complexes by human TP53 antibody was subjected to SYBR Green PCR with primers containing potential TP53 binding elements from different regions in the promoter of indicated cytokines. The PCR product levels of Input DNA and IgG were used as positive and negative controls respectively. Data represents 2 biological and 2 technical repeats.

**Figure 7:**
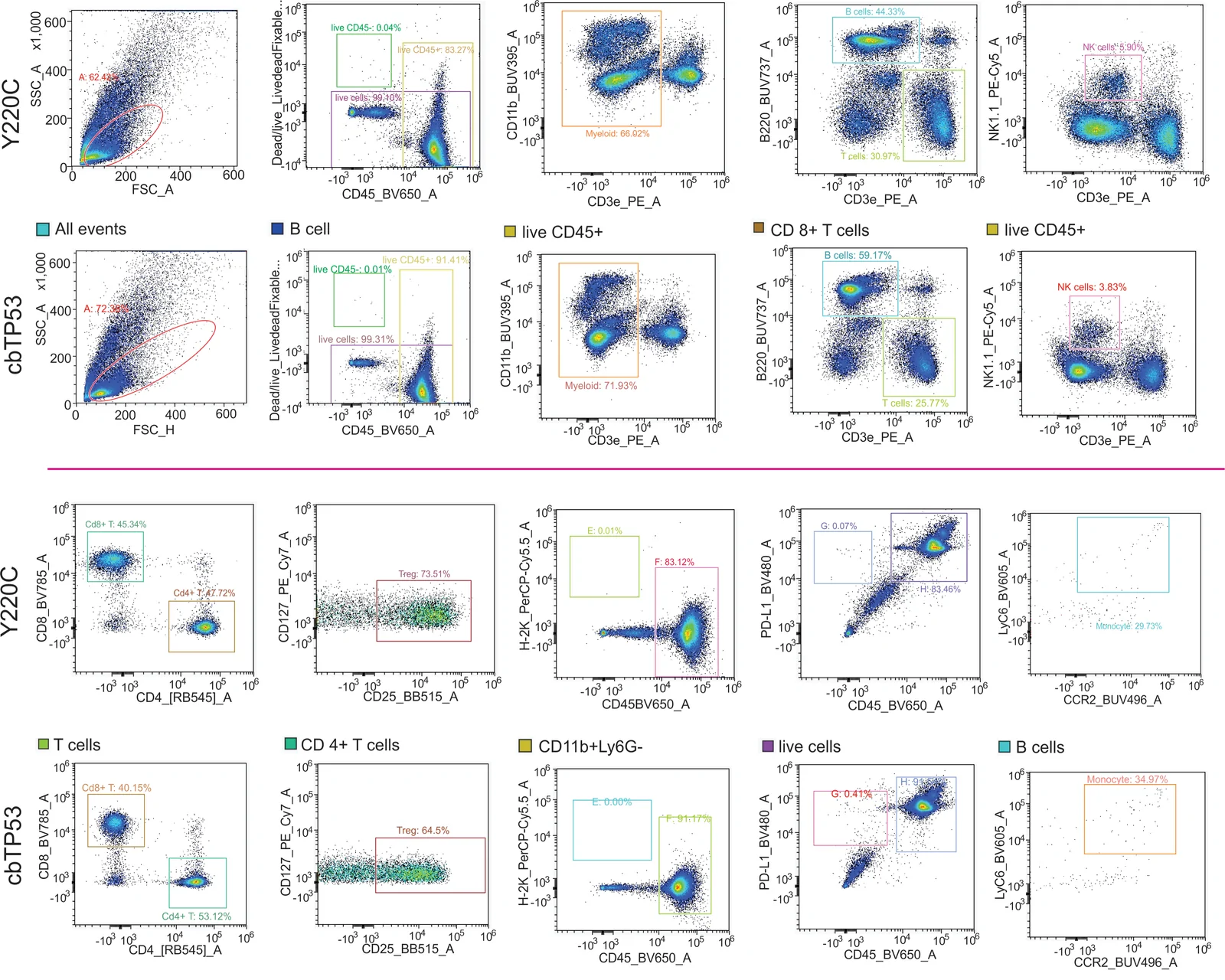
** The cbTP53 and Y220C own different oncogenic properties in mice.** Multiparametric flow cytometry analyses on the fresh whole blood after red blood cell lysis characterizing immune cells subsets in Y220C (n = 5 mice/group; pooled blood) and cbTP53 (n = 7 mice/group; pooled blood) mice. Dot plots for each immune cell subset. Gates within each plot indicate cell subset and corresponding frequency within viable positive cells. Examples of parent gates are shown; frequencies were calculated using the specific gating strategies.
