# Supplementary material for: Combined Point Mutation in a Single Allele of the TP53 Gene Potentiates the Oncogenic and Transcriptional Properties of TP53 in Lung Cancer
Source: Int J Biol Sci. 2026 Sep 10;22(15):8139–57. doi: 10.7150/ijbs.131687 (PMC13685541; doi:10.7150/ijbs.131687)
Supplement: Supplementary file 1 [file ijbsv22p8139s1.pdf]

Combined Point Mutation in a Single Allele of the TP53 Gene Potentiates the Oncogenic and Transcriptional Properties of TP53 in Lung Cancer

Sicheng Bian, Huiqin Bian, Hiroaki Koyama, Dmytro Kompaniiets, William Tse, Mithun V Shah, Aref Al-Kali, Alexander Miron, Bin Liu, Shujun Liu

Supplemental Figures and Legends 1-7

Supplemental Table 9

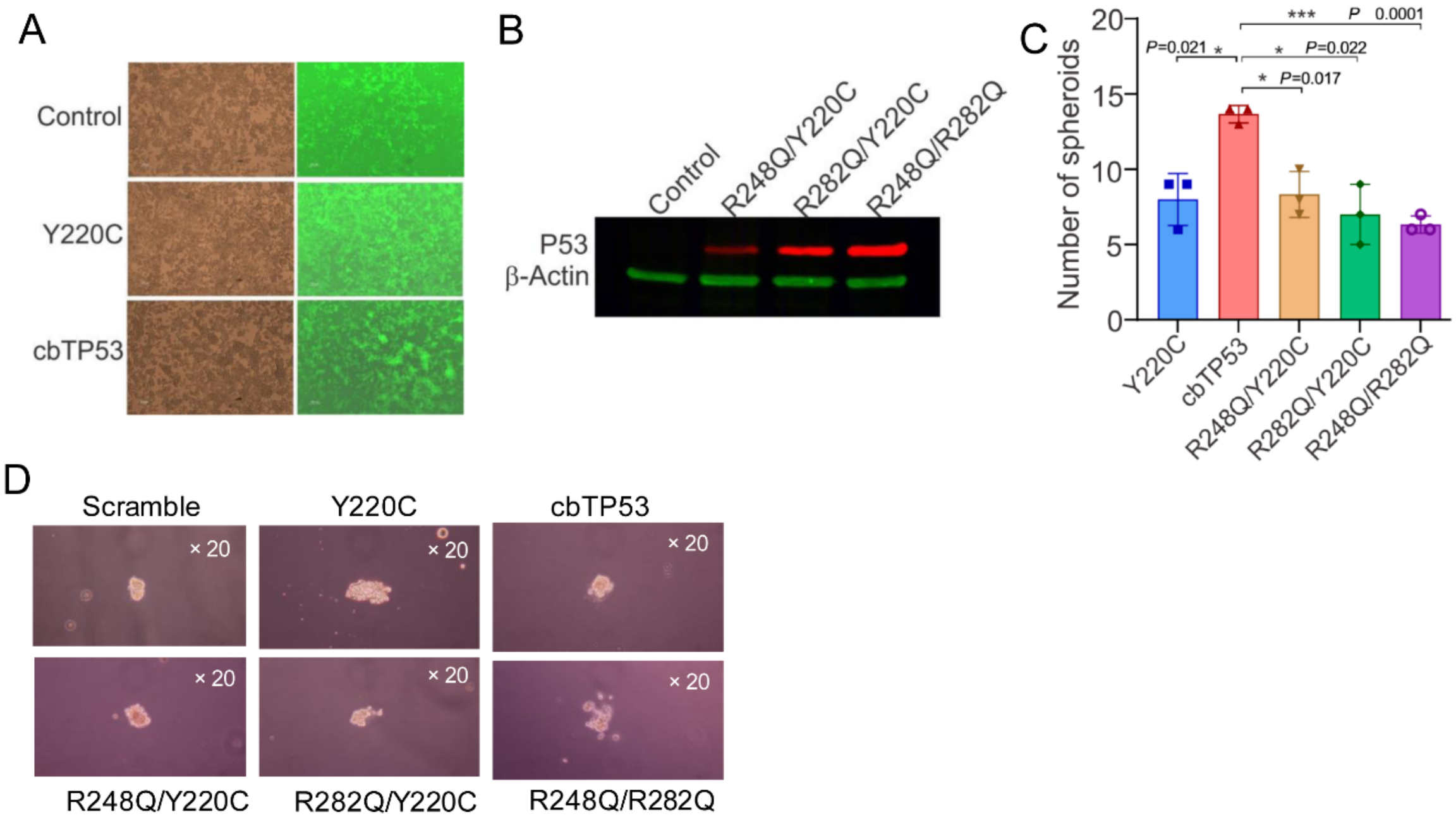

Supplementary Figure 1. Lentiviral transduction efficiency, p53 expression, and spheroid formation in LLC/2 cells. LLC/2 cells were transduced with lentiviruses expressing Y220C, cbTP53, or a scrambled control. At 72 hours post-infection, GFP-positive cells were isolated via two rounds of fluorescence-activated cell sorting (FACS) and subsequent puromycin selection (2 μg/ml). (A) Representative brightfield (left) and fluorescence (right; excitation/emission wavelength: 475 nm) micrographs of transduced cells. (B) Western blot analysis of p53 protein expression in overexpressing LLC/2 cells. Data are representative of three independent experiments. (C, D) Spheroid formation assays of LLC/2 cells expressing the indicated p53 variants. Representative images of spheroids captured on day 11 (C) and corresponding quantification (D). Data are presented as mean ±SD.

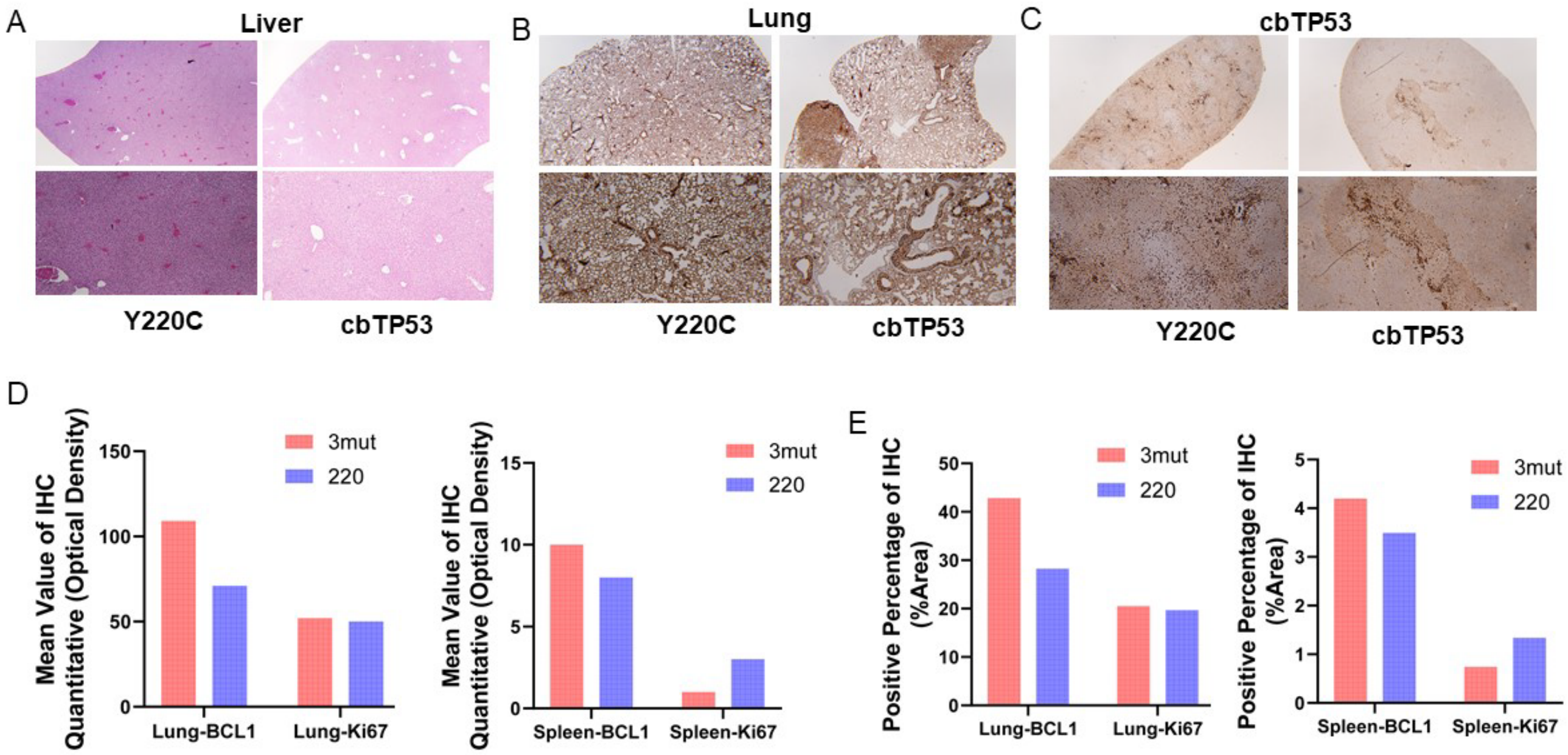

Supplementary Figure 2. Histopathological examination of organs derived from cancer-bearing mice. (A) Representative images of H&E staining of livers (n = 3/group). (B, C) Representative images of IHC staining for lungs (Cyclin D1/BCL1, B) or spleens (Ki-67, C) (N = 3/group). (D, E) Quantification of Ki-67 or BCL1 signals in lungs and spleens (n = 3/group). Note: 220 = Y220C; 3 mut = cbTP53.

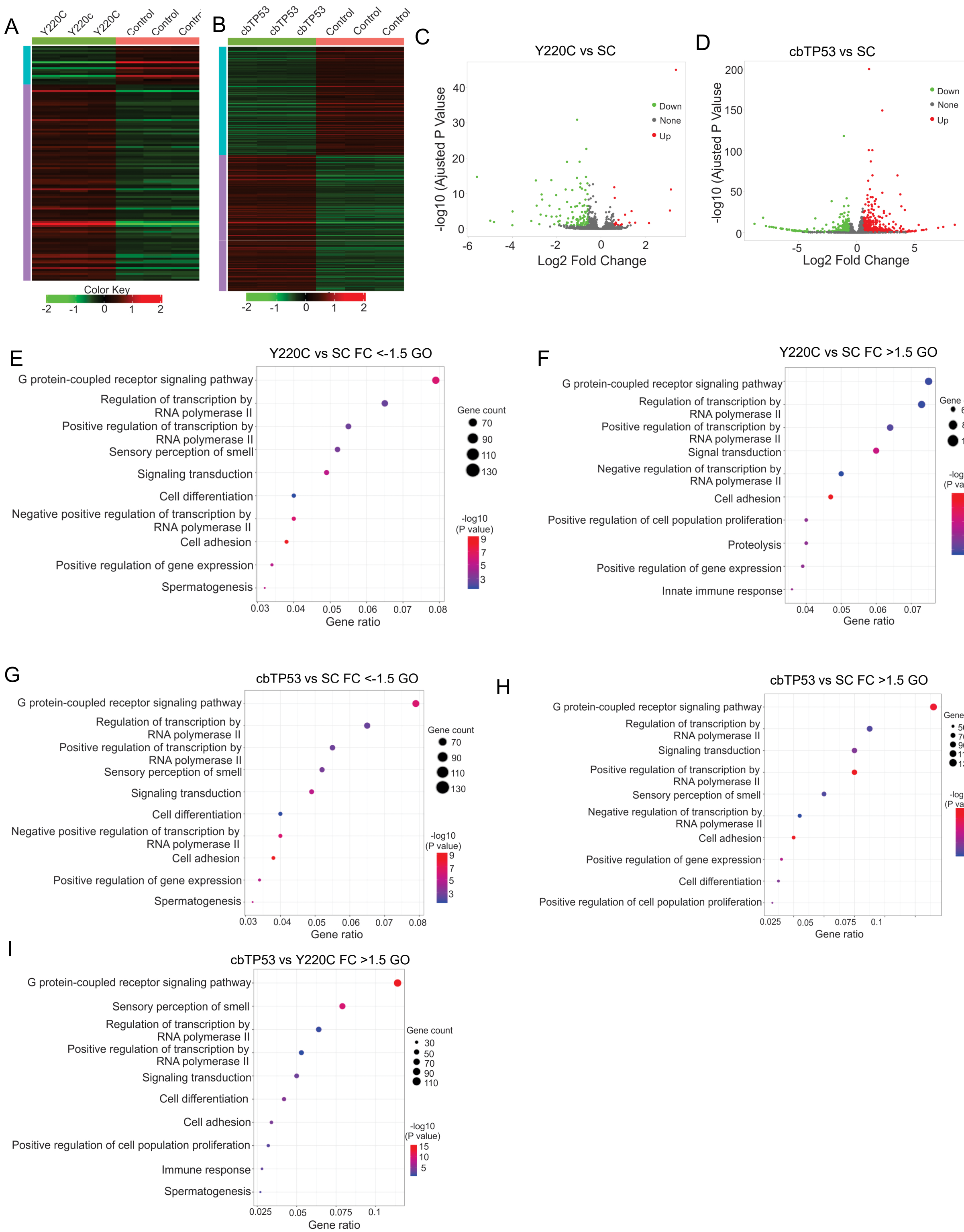

Supplementary Figure 3. Comparative analysis of RNA-seq data reveals distinctive transcriptional profiles in LLC/2 cells expressing cbTP53 or Y220C. (A and B) Heatmaps of

the differentially expressed genes (DEGs) in Y220C vs control/scramble (A) or cbTP53 vs control/scramble (B). (C and D) Volcano plot showing the differentially expressed genes in Y220C (C) or cbTP53 (D) comparing to scramble (SC) control. (E-I) Gene set enrichment analysis of up- (FC >1.5) and down- (FC <-1.5) regulated genes for GO terms in Y220C vs control/SC (E, F), cbTP53 vs control/SC (G, H) or cbTP53 vs Y220C (I). The top 10 enriched GO terms are shown. The color and size of dots represent *P* value and gene number assigned to the corresponding GO terms. Note: GO, Gene Ontology; FC, fold change.

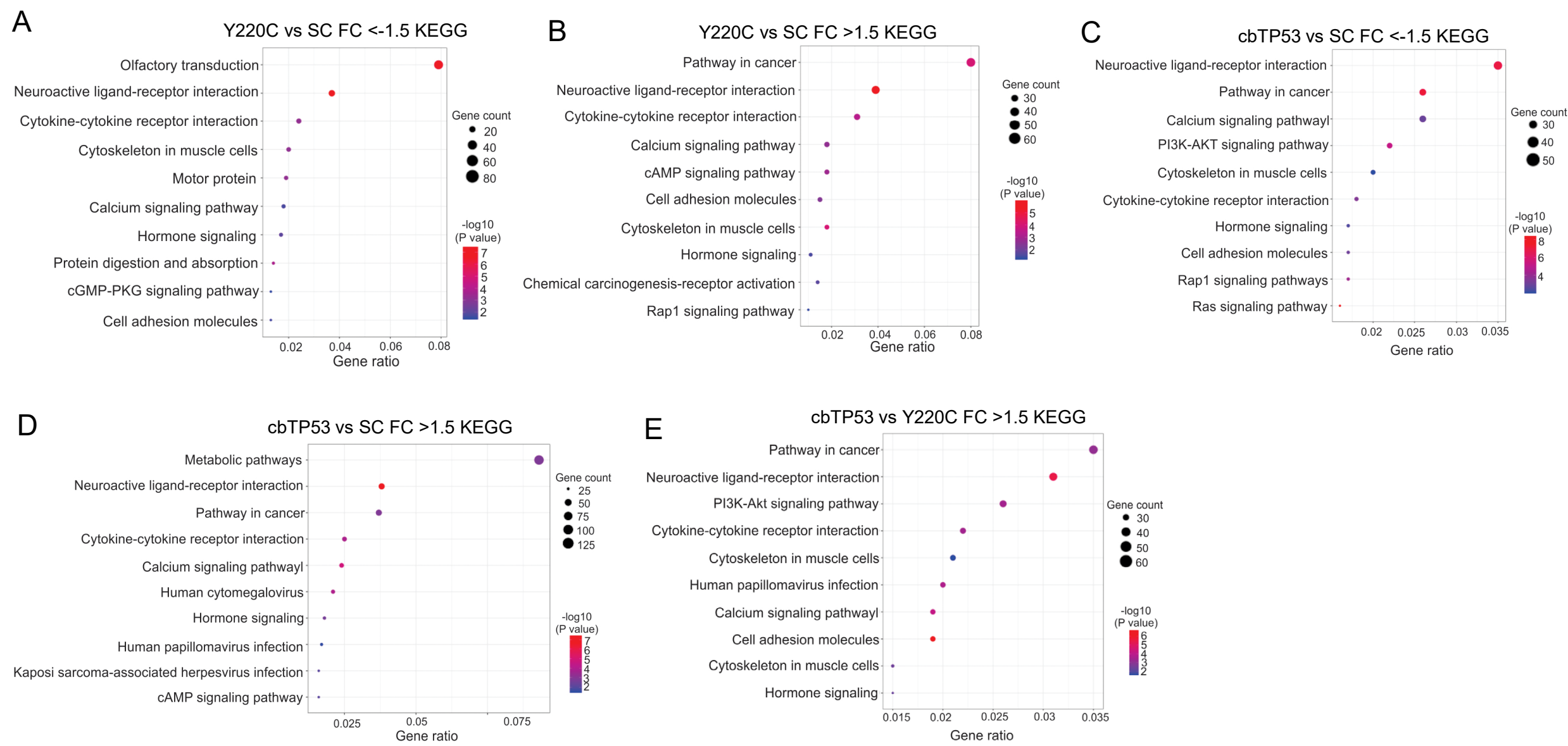

Supplementary Figure 4. Analyses of RNA-seq data. (A-E) Functional annotation of the DEGs by KEGG enrichment analyses. GeneRatio reflects the gene number ratio in each KEGG pathway in various comparisons Y220C vs SC (A, B), cbTP53 vs SC (C, D) or cbTP53 vs Y220C (E). The color and size of dots represent *P* value and gene number assigned to the corresponding KEGG pathway. The top 10 enriched KEGG pathways are shown.

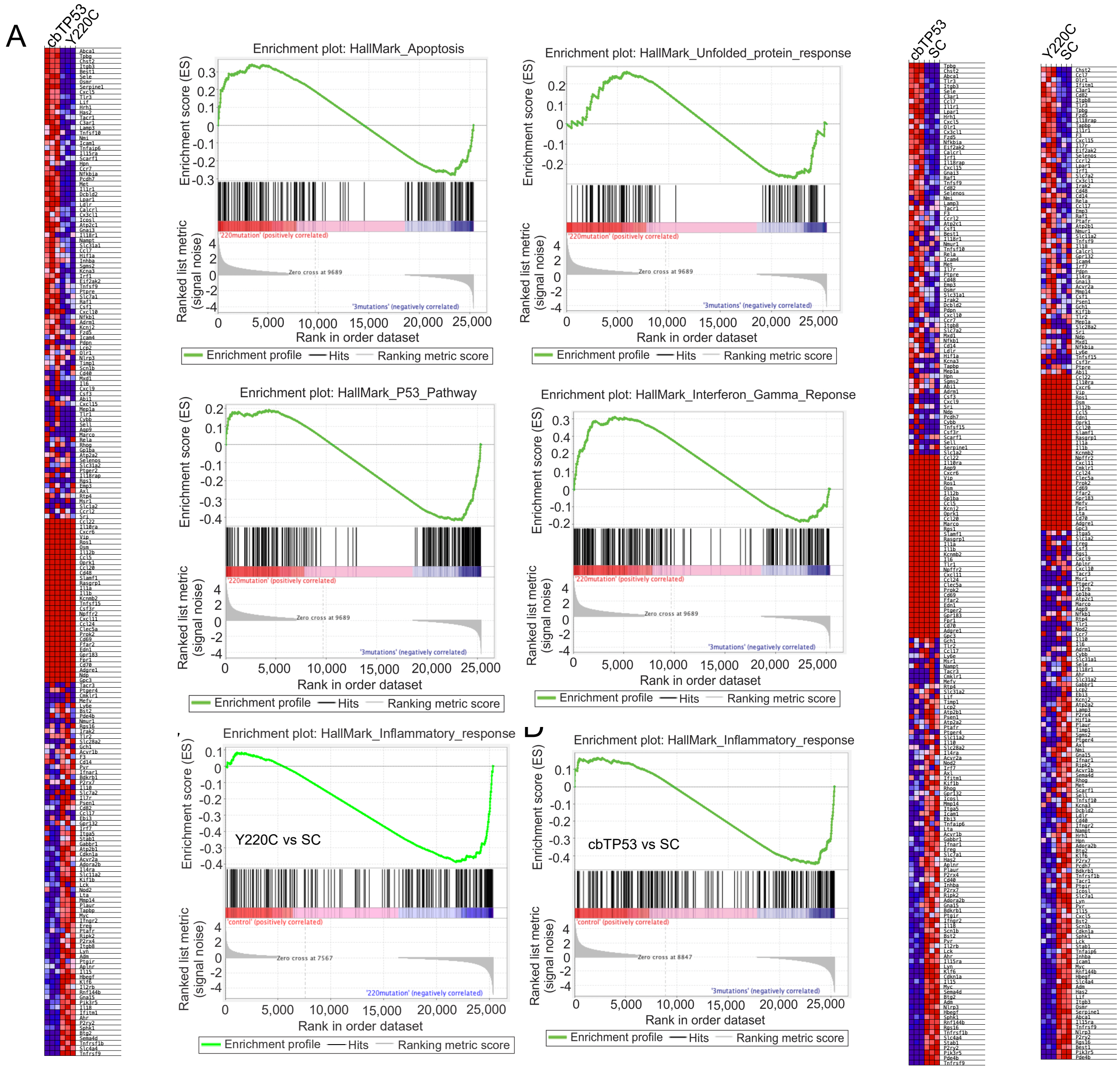

Supplementary Figure 5. Hallmark gene set analyses from all differentially expressed genes. (A) Heat map from GSEA (reported in left panel of Figure 5G) showing the core-enriched genes in inflammatory response. (B-D) GSEA enrichment score plots showing significant correlation between genes associated with different hallmark pathways and DEGs in cbTP53 vs Y220C, Y220C vs SC (C) or cbTP53 vs SC (D). (E and F) Heat maps from GSEA showing the core-enriched genes in inflammatory response. GSEA, Gene Set Enrichment Analysis; DEGs, differentially expressed genes; SC, scramble.

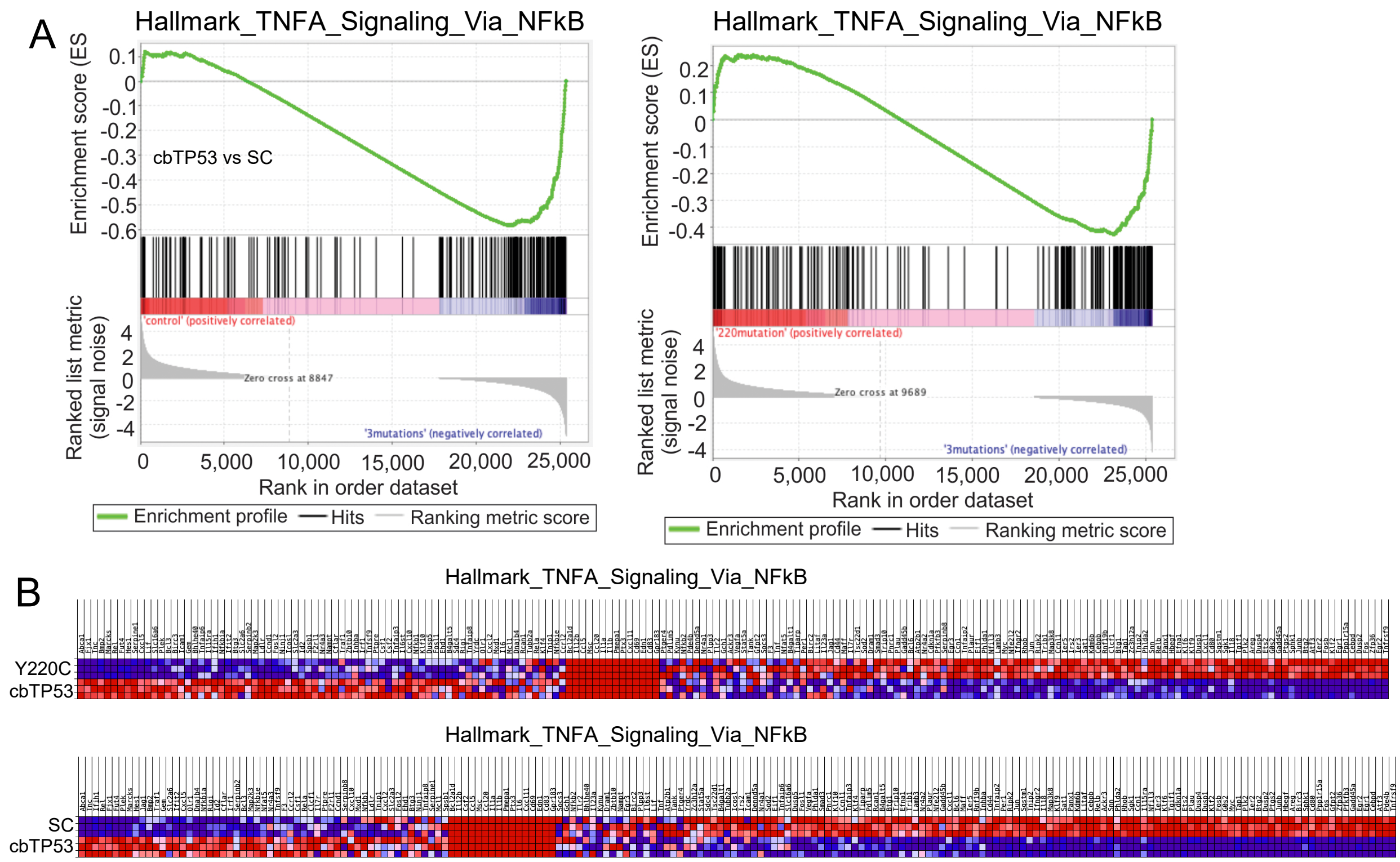

Supplementary Figure 6. Gene set enrichment analysis for all differentially expressed genes. (A) GSEA enrichment score plots showing significant correlation between genes associated with TNFA signaling via NFkB pathway and DEGs in cbTP53 vs SC (left) or cbTP53 vs Y220C (right). (B) Heat maps from GSEA in A showing the core-enriched genes in TNFA signaling via NFkB pathway. GSEA, Gene Set Enrichment Analysis; DEGs, differentially expressed genes; SC, scramble.

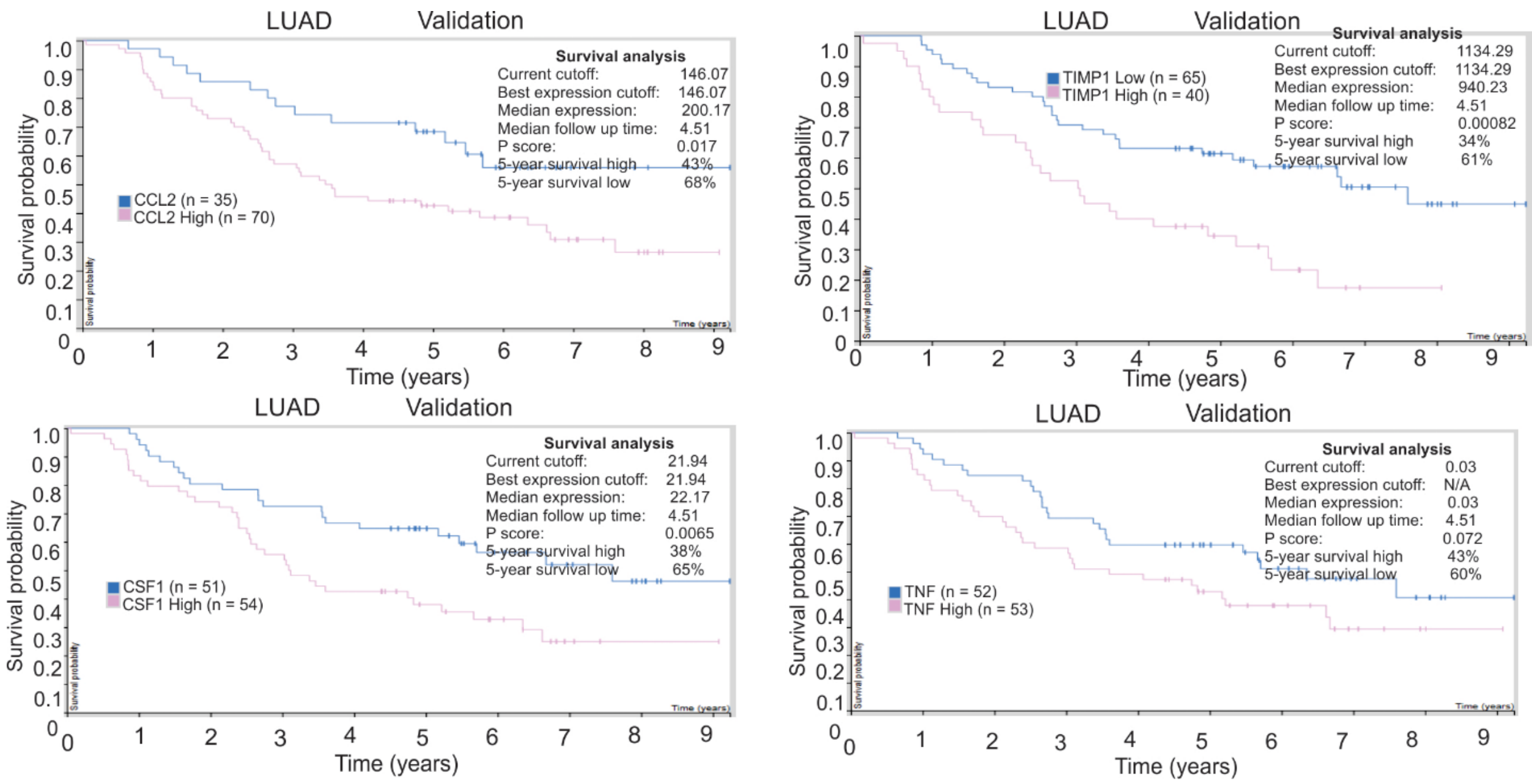

Supplementary Figure 7. Survival analysis of TCGA datasets for prognostic potentials of the detected cytokines (overall survival).

Supplementary Table 9. Sequence of qPCR primers used for gene expression

	Name		Oligo sequences (5' to 3')
qPCR	TNF-α NC_000083.7:c35422983-35420983	forward	CTGAACTTCGGGGTGATCGG
		reverse	GGCTTGTCACTCGAATTTTGAGA
	M-CSF NC_000069.7:c107670048-107668048	forward	GTGTCAGAACACTGTAGCCAC
		reverse	TCAAAGGCAATCTGGCATGAAG
	IFN-γ NC_000076.7:118274951-118276951	forward	ACAGCAAGGCGAAAAAGGATG
		reverse	TGGTGGACCACTCGGATGA
	CXCL2 NC_000071.7:91049758-91051758	forward	CCAACCACCAGGCTACAGG
		reverse	GCGTCACACTCAAGCTCTG
	TIMP-1 NC_000086.8:20734524-20736524	forward	GCAACTCGGACCTGGTCATAA
		reverse	CGGCCCGTGATGAGAAACT
	CXCL1 NC_000071.7:91037104-91039104	forward	ACTGCACCCAAACCGAAGTC
		reverse	TGGGGACACCTTTTAGCATCTT
	CCL2 NC_000077.7:81924403-81926403	forward	TAAAAACCTGGATCGGAACCAAA
		reverse	GCATTAGCTTCAGATTTACGGGT
		reverse	ATTCGAAGGAGCAGCACACT
